# Evaluating the Slow-Release Fertilizer Effects of Calcium Potassium Phosphates from Mechanochemically Treated Calcium Carbonate Materials with KH_2_PO_4_

**DOI:** 10.1021/acsomega.5c12414

**Published:** 2026-10-17

**Authors:** Roger Borges, Amanda S. Giroto, Stella F. Valle, Benedict Ohrem, Tainára Orlando, Paloma Neves de Oliveira, Nicolai D. Jablonowski, Caue Ribeiro

**Affiliations:** † Federal University of Technology – Paran, 85884-000 Medianeira, PR, Brazil; ‡ Federal University of São Paulo, Science and Technology Institute, 12231-280 São José dos Campos, SP, Brazil; § Institute of Bio- and Geosciences, IBG-2: Plant Sciences, 28334 Forschungszentrum Jülich GmbH, 52425 Jülich, Germany; ∥ Lorena School of Engineering (EEL), University of São Paulo (USP), 12602-810 Lorena, SP, Brazil; ⊥ Embrapa Instrumentation, XV de Novembro Street, 1452, 13560-970 São Carlos, SP, Brazil

## Abstract

Developing slow-release fertilizers, carefully adjusting their solubility profiles, is an important way to improve nutrient use efficiency and potentially reduce environmental impacts associated with nutrient losses and accumulation in water bodies, such as potential eutrophication. In this work, CaCO_3_ from industrial byproducts (spent lime, i.e., carbokalk, and eggshells) was mechanochemically activated with KH_2_PO_4_ and citric acid as an additive. X-ray diffraction indicated the formation of calcium potassium phosphate phases, along with residual CaCO_3_, depending on milling conditions. However, citric acid addition suppressed crystalline phosphate formation, likely influencing species organization and favoring poorly crystalline or amorphous phosphate phases, possibly through Ca complexation. Phosphorus percolation from milled samples remained low at early stages (0.2–4% after 2 days) and reached only 21–35% after 7 days, whereas potassium showed faster release, with 0.2–10% at 2 days and up to ∼100% percolation after 7 days. Greenhouse trials with *Brachiaria brizantha* over three harvest cycles suggested improved nutrient management, evidenced by a more gradual biomass increase and nutrient uptake, in contrast to the early peak and subsequent decline observed in positive controls using KH_2_PO_4_ and triple superphosphate (TSP). Thus, mechanochemical processing offers a smarter product capable of gradually releasing its fertilizer content, offering a promising route toward efficient and sustainable fertilization strategies.

## Graphical abstract

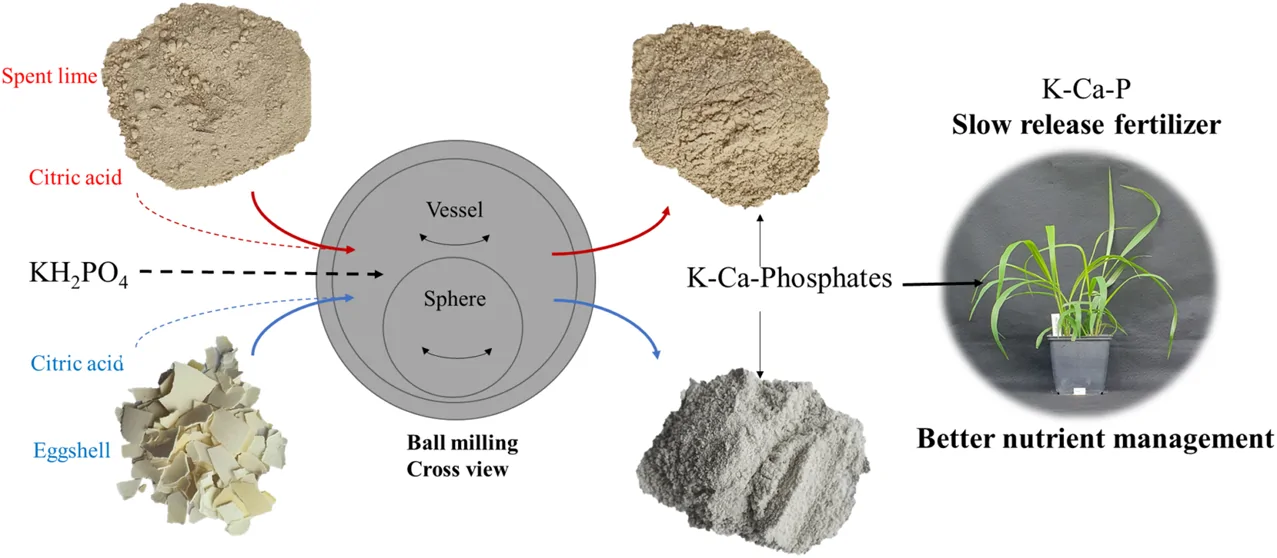

## Introduction

It is expected that the world population will exceed 10 billion by 2050, posing an important issue for global agriculture.^38,39^ Thus, the development of more sustainable fertilization strategies will be essential, particularly those related to keeping nutrients in surface layers and making them available to plants, potentially minimizing nutrient leaching into groundwater while reducing the need for excessive fertilization. Phosphorus (P) is one of the most important macronutrients for plant growth, playing a central role in photosynthesis and energy transfer.^1^ Monopotassium phosphate (KH_2_PO_4_) is an interesting option for P fertilization as it additionally provides potassium (K) to plants, another macronutrient that is involved in enzyme activation and cell growth.^2^ Moreover, soil K strengthens crop resistance to drought, pests, and diseases.^3^ However, KH_2_PO_4_ is a highly water-soluble fertilizer, i.e., easily leached into nearby water bodies, which can promote eutrophication and other environmental problems.^4,5^ Developing smart strategies to control its solubilization and release can reduce environmental impacts, nutrient, and economic losses, while also enhancing fertilizer efficiency through a nutrient supply according to crop demands.^6,7^

Another important aspect of greener fertilizer production is the use of cost-effective and environmentally friendly raw materials and manufacturing processes. Among the available alternatives, polymer-coated formulations and struvite stand out. While polymer-coated fertilizers provide strong short-term control over nutrient release, mineral-based systems may offer advantages in terms of soil compatibility, absence of microplastic residues, and alignment with circular-economy strategies.^43^ Although struvite is considered a promising candidate for slow-release fertilization and has been investigated in previous studies by our group,^42^ its increasing demand highlights the need for the development of additional mineral-based fertilizers with tunable release properties. In this context, mechanochemical activation emerges as a green chemistry approach that employs mechanical energy to induce chemical transformations, enabling the preparation of mineral fertilizers with modulated nutrient release.^8–13^ Mechanochemical processes are sometimes associated with relatively high energy demand. However, Brekalo et al.^40^ reported that energy use decreases from 7.6 W·h·g^–1^ at small scale to approximately 3–4 W·h·g^–1^ at pilot scale, with continuous processes offering the best performance and highest space–time yields. These results indicate that mechanochemical synthesis becomes substantially more energy-efficient as the production scale increases. Moreover, as a solvent-free solid-state process, it reduces waste generation and the need for multiple preparation steps. This is particularly important when using residue feedstocks. The mechanochemical process can lead to advanced fertilizers, such as calcium and potassium phosphates (K_2_CaP_2_O_7_ and K_3_CaH­(PO_4_)_2_), which can be obtained by phosphate reactions with lime (KH_2_PO_4_, K_2_HPO_4_, or Ca­(H_2_PO_4_)_2_·H_2_O). By modifying KH_2_PO_4_ structure with calcium (Ca), the produced material was able to provide a more gradual nutrient release than the precursor, showing great potential to be used as a slow-release fertilizer.^11^ Ca bioavailability can be improved with this strategy as well. Besides its role as a macronutrient, Ca can also benefit the soil as a conditioner, reducing soil acidityand improving soil structure.^14,15^ However, these materials remain scarcely explored in applied studies, e.g., pot trials in controlled conditions, where their influence on plant development and their dynamics within soil–plant systems can be revealed in a comprehensive investigation.

Thus, the present work aims to evaluate the agronomic performance of different eco-friendly fertilizers based on K and Ca phosphates obtained via mechanochemistry. Similar to our previous study, eggshells were used as a natural nontoxic calcite source material and an abundant waste from the food industry,^16^ also reported to be contributing positively to the remediation of soils contaminated with heavy metals.^44^ Spent lime from the sugar beet processing (so-called carbokalk) industry was also investigated as a cheap CaCO_3_-based byproduct material.^17^ This specific spent lime (carbokalk) from the sugar beet industry is already commercialized and routinely applied in agriculture as a liming material, which indicates its suitability and safety for soil use.^41^ Accordingly, two hypotheses were tested in this study: (i) that citric acid addition can modulate CaCO_3_ reactivity during mechanochemical processing, thereby influencing phosphate phase formation and/or nutrient availability; and (ii) that the resulting materials would provide a more gradual supply of P and K to plants compared to highly soluble fertilizers, leading to sustained nutrient uptake over successive harvests. The tropical forage grass *Brachiaria brizantha* was selected as a model plant due to its P and K demand and suitability for consecutive harvest cycles.^13,18^

## Materials

Commercial monopotassium phosphate (KH_2_PO_4_, brand Roth) was used as the primary precursor. Calcium carbonate (CaCO_3_) was obtained from two alternative sources: (i) spent lime (Carbokalk) provided by the sugar beet processing industry Pfeifer & Langen GmbH & Co. KG - Jülich, Germany (<0.3 mm, 50 mesh), and (ii) eggshells collected from a local food industry, thoroughly washed, dried, milled, and sieved prior to use (∼3 mm, 6 mesh). Citric acid (CA) (brand Roth) was employed as an additive in selected synthesis batches to evaluate its effect on phase formation and nutrient release. Water-soluble triple superphosphate (TSP, 45.0% P_2_O_5_, containing 40.0% water-soluble phosphate) and KH_2_PO_4_ were used as positive controls in percolation and greenhouse experiments.

## Methods

### Mechanochemical Synthesis

The formation of calcium and potassium phosphates can be efficiently achieved by mechanochemical processes. Recently, we demonstrated that mechanochemical treatment of CaCO_3_ enhances its reactivity to efficiently react with KH_2_PO_4_.^11^ There is also the formation of hydroxyapatite during the process of nutrient release. On the basis of that, two sets of experiments were carried out with the two proposed CaCO_3_-based materials, eggshells and carbokalk. Each material was milled individually with KH_2_PO_4_ while maintaining a fixed Ca:K:P molar ratio of 1.25:1:1. The same milling procedure and molar ratio were applied but with the addition of 2.5 mmol of citric acid (CA) to the mixture. This second milling strategy was designed to evaluate the effect of citric acid on both milling processes and the nutrient release in subsequent greenhouse applications.

The mechanochemical processes were conducted using a BM500 Anton Paar ball mill, a stainless steel vase, and one stainless steel sphere, at 30 Hz, with a milling time ranging from 20 to 140 min. The schematic representation of the mechanochemical process is illustrated in Figure 1.

**Figure 1:**
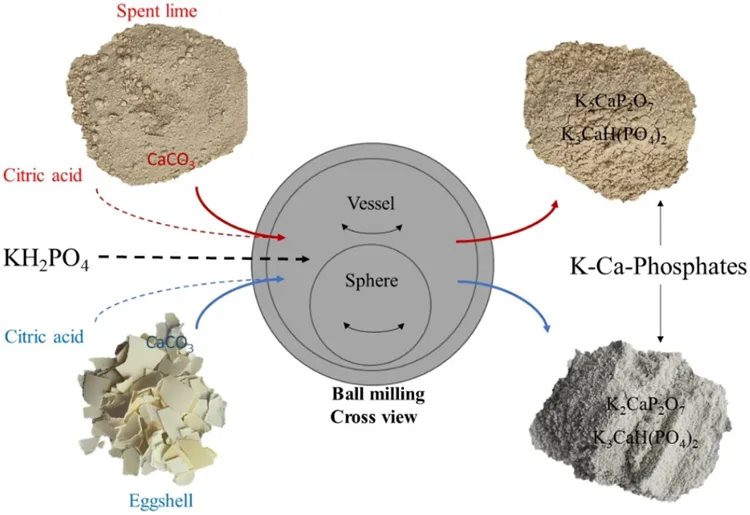
Schematic representation of the mechanochemical process.

### Characterization

In order to evaluate the chemical transformation from the mechanochemical process, all the precursors and samples after milling were characterized by different techniques. The crystalline phases were qualitatively identified by X-ray diffraction (XRD) analyses using an XRD-6000 diffractometer (Shimadzu). The relative intensity of the diffraction peaks was registered in the angular range (2θ) of 10–70°, using a Cu Kα incident beam (λ = 0.15406 nm) and a scanning speed of 2° min^–1^ at 30 kV voltage and 30 mA current. TGA-Q500 TA Instruments equipment was used to analyze the thermogravimetric behavior under a 100 mL min^–1^ N_2_/O_2_ atmosphere; samples were placed in alumina crucibles and heated from ∼20 to 900 °C (10 °C min^–1^). The functional groups of the synthesized materials were analyzed by Fourier-transform infrared spectroscopy (FTIR) using a Vertex 70 spectrometer (Bruker) equipped with an attenuated total reflectance (ATR) accessory. Spectra were collected in the range of 4000–400 cm^–1^, with 32 scans per sample and a resolution of 2 cm^–1^. Carbon speciation and possible organometallic interactions in the synthesized materials were investigated by solid-state ^13^C magic angle spinning nuclear magnetic resonance (MAS NMR) using an Avance III HD spectrometer (Bruker). Samples were spun at 10 kHz and analyzed using the ^13^C cross-polarization magic angle spinning (CP/MAS) sequence, with 2048 scans, an acquisition time of 40 ms, a contact time of 4.5 ms, and a recycle delay of 5 s. Chemical shifts were referenced to hexamethylbenzene at 17.3 ppm. Phosphorus speciation was evaluated by solid-state ^31^P MAS NMR using the same instrument. Spectra were acquired at a spinning rate of 10 kHz using a high-power decoupling (HPDec) sequence. Chemical shifts were referenced to adenosine diphosphate (ADP), with the phosphate signal set at 0.8 ppm.

### Determination of Neutralizing Power (NP) and Total Neutralizing Power (TNP)

The starting materials, Carbokalk and eggshells, are CaCO_3_-based materials known to be effective for correcting soil acidity, depending on particle size. After the mechanochemical process, it was expected that the CaCO_3_ content would be significantly reduced over time; however, it was believed that residual CaCO_3_-based precursors could maintain some percentage of neutralizing power. The methodology applied is the same as that adopted for commercial limestone. Briefly, 50 mL of standardized 0.5 M HCl was added to 0.5 g of each sample, and the mixture was gently boiled for 5 min. After cooling, the volume was adjusted to 100 mL with deionized water and filtered. 50 mL of the supernatant was titrated with a standardized 0.25 M NaOH solution, using phenolphthalein as an indicator. The eggshell precursor was previously pulverized to determine neutralizing power (NP). The NP was determined by noting the volume of NaOH solution used, employing the following formula:

$$NP=\frac{\left(V_{HCl}\times M_{HCl}\right)-\left(V_{NaOH}\times M_{NaOH}\right)}{m_{sample}}\times f$$

*V*_HCl_ = volume of standardized HCl added (L), *M*_HCl_ = molarity of HCl (mol·L^–1^), *V*_NaOH_ = volume of standardized NaOH used in the back-titration (L), *M*_NaOH_ = molarity of NaOH (mol·L^–1^), *m*_sample_ = mass of the sample (g), *f* = conversion factor to express NP in the desired units (e.g., % CaCO_3_ equivalent).

### Nutrient Percolation in Sand Soil Substrate

The investigation of the fertilizer species percolation in soil provides insight into the tendency of these materials to either remain in the upper soil layers, where they are readily available to plant roots, or to leach into deeper layers. The proposed method was adapted from the literature.^20,21^ Briefly, PVC tubes measuring 25 cm in height and 3.4 cm in inner diameter were used, and each tube was filled with 0.160 dm^3^ of washed sand. Then, the appropriate amount of each sample was added, corresponding to 200 mg of P dm^–3^, and covered with another 0.020 dm^3^ of sand, totaling a column of 0.180 dm^3^ (20 cm) of sand. All experiments were conducted in triplicate (*n* = 3). The system was maintained for 7 days with 100% water saturation, with 10 mL of deionized water added each day, simulating an intense rain season. This approach was selected deliberately to stress test nutrient retention and release behavior. After percolation experiments, the solid residual samples were collected and characterized by XRD to evaluate the possible crystallographic changes. To corroborate the percolation results, preliminary laboratory simulation experiments were conducted to evaluate the nutrient release behavior of the synthesized materials in water at contact times of 30 min, 1 day, and 5 days. After each contact period, the leachates were collected and analyzed for P, K, and Ca by ICP-OES.

The percolation data obtained for all treatments were fitted to commonly applied kinetic release models to more accurately describe and compare the solubilization behaviors of the materials. These kinetic models provide a mathematical description of the release behavior, but they do not imply a specific mechanistic pathway. In this approach, the cumulative nutrient release profiles were evaluated using zero-order, Higuchi, pseudo-first-order, and pseudo-second-order kinetic models, enabling a detailed assessment of the release mechanisms and their differences among the tested samples.

### Greenhouse Application

Greenhouse experiments with the final milled samples were conducted to assess their fertilizing behavior in terms of Ca, K, and P supply. These experiments were carried out over a total period of four months under semicontrolled greenhouse conditions at the Institute of Bio- and Geosciences, IBG-2: Plant Sciences, Forschungszentrum Jülich GmbH, 52425 Jülich, Germany 50°54′35.0″N 6°24′47.4″E. *Brachiaria brizantha* was selected as the test crop due to its agronomic relevance and well-documented nutrient uptake characteristics.^22,23^ This grass is widely cultivated in tropical and subtropical regions, particularly in pasture systems, and plays a critical role in soil conservation and livestock production.^24^ Its extensive root system and high biomass production make it an efficient model for evaluating nutrient availability in soil. Previous studies have established *Brachiaria brizantha* as a model plant for phosphorus demand,^18^ given its sensitivity to P availability and capacity to respond to fertilization. More recently, it has also been recognized as a model for potassium demand.^19^

Two concentrations of P were evaluated in this study, 100 and 200 mg of P dm^–3^. The milled product samples are described as follow: sample code: 1, Carbokalk+KH_2_PO_4_; sample code: 2, Carbokalk+KH_2_PO_4_+CA; sample code: 3, Eggshel+KH_2_PO_4_; sample code: 4, Eggshel+KH_2_PO_4_+CA Monobasic potassium phosphate (MKP–sample code: 5) and triple superphosphate (TSP–sample code: 6) were used as positive controls, owing to their high *P* availability, whereas Bayovar rock (sample code: 7) served as negative control due to its very low *P* availability (negative control). A nonfertilized control was employed as a total negative reference (sample code: 8). Additional comparative controls were also evaluated: physical mixture of Carbokalk+KH_2_PO_4_ without milling (sample code: 9); and physical mixture of Eggshel+KH_2_PO_4_ without milling (sample code: 10). Nutrient concentrations were calculated based on the nutrient content of the respective samples and controls (Table 1S).

Approximately 2 kg of sand was used as substrate in plastic pots, with five replicates per treatment. Phosphorus was supplied by the respective samples and controls to achieve the target concentrations of 100 or 200 mg P dm^–3^. To avoid nutritional limitations unrelated to phosphorus availability, all other macro- and micronutrients were adjusted and standardized across the fertilized treatments. Basal fertilization consisted of 100 mg of N dm^–3^, as urea, 40 mg of Mg dm^–3^, and 50 mg of S dm^–3^, as magnesium sulfate, 150 mg of K dm^–3^, and 200 mg of Ca dm^–3^, as KCl and CaCO_3_, respectively, when their concentrations in the samples and controls were not sufficient, applied uniformly to all treatments to avoid nutritional deficiencies. It should be noted that the basal application of K and Ca, although necessary to avoid nutrient deficiencies, may partially mask the treatment-related differences in K and Ca release. Therefore, the observed plant responses are primarily interpreted in terms of phosphorus availability. These fertilization rates follow standard agronomic recommendations and are within ranges that do not disrupt the soil base balance or increase the risk of soil compaction.

The plant experiments were carried out under semicontrolled greenhouse conditions. Throughout the entire duration of the experiment, the average air temperature was 19.7 ± 3.1 °C, and the average humidity was 51.8 ± 12.0%. Artificial light supplementation was used for 12 h during the day. After fertilizer application to the pots, five *Brachiaria brizantha* seeds were sown per pot, keeping two plants per pot after 3 weeks. To avoid drought stress, the plants were regularly watered, maintaining approximately 50% of the maximum water holding capacity (WH*C*_max_), and after 4 weeks, each pot was fertilized with micronutrients solution, according to Table 2S.

The total shoot and leaf biomass was harvested three times during the greenhouse experiment, first after 8 weeks, followed by 12 and 16 weeks after seeding, cutting the plants 5 cm above the soil surface. At the third and final harvest, the roots were also collected and carefully separated from the substrate by thorough washing to remove any adherent particles.

All shoot and root biomasses were dried at 60 °C, and their dry weight was determined. Soil substrate aliquots of each treatment were also collected and dried at 60 °C. After drying, all biomasses and substrate aliquots were homogenized by milling using a Retsch MM400 ball mill (Germany) at 30 Hz for 1 min. Then, approximately 50 mg of each sample was digested in closed vessels in a microwave system with 1 mL HNO_3_/0.5 mL H_2_O_2_. The vessels were heated to 160 °C within 4 min and held at this temperature for 3 min. Each digestion solution was transferred and made up to a total volume of 50 mL, and analyzed by ICP-EOS, Thermo Scientific iCAP 7600 (Germany). The uptake of P, Ca, and K was estimated based on their accumulated content (%) in the dry shoot and root matter and the total plant dry matter produced (mg). The data were analyzed using ANOVA, and treatment means were compared with Tukey’s test at a 5% significance level.

### Statistical Analysis

Statistical analyses were performed using factorial ANOVA, considering treatments and harvests as fixed factors and including their interaction, for each phosphorus dose. When significant effects were detected, mean comparisons were conducted using Tukey’s HSD test at the 5% significance level. All analyses were carried out in Python using the Statsmodels package in a Google Colab environment. Biomass measurements across sequential harvests were obtained through destructive sampling, such that different plants (independent biological replicates) were collected at each time point. Therefore, observations do not represent repeated measurements on the same experimental units, and the assumption of within-subject correlation required for repeated-measures analysis is not met.

## Results and Discussion

### Characterization

The mechanochemical reaction between CaCO_3_ and KH_2_PO_4_ was confirmed by XRD (Figure 2). For the materials without the addition of citric acid, a new phase was observed after 20 min of milling, and it is probably associated with K_2_CaP_2_O_7_ formation, potassium calcium pyrophosphate (PDF: 50-1563). However, the formation of other amorphous (hydrogen) calcium potassium phosphates cannot be excluded, for instance, K_3_CaH­(PO_4_)_2_,^11^ or even disordered Ca–K phosphate phases. The characteristic peaks of KH_2_PO_4_ were no longer observed after 40 min of milling;^25^ however, the CaCO_3_-related peaks were apparently not fully consumed.^26^ Even after 120 min of milling, the intense CaCO_3_ peak at 29.5° transformed into a broadened shoulder, making it difficult to distinguish from the adjacent peak at 30.0°, which corresponds to the newly formed phosphate phase. However, a residual amount of calcium carbonate gives the final product some potential for pH modulation of acidic soils. The residual content of CaCO_3_ can be adjusted, as the XRD data indicate that varying the milling time allows control over the remaining CaCO_3_: shorter times result in higher amounts, and longer times in lower amounts of residual CaCO_3_.

**Figure 2:**
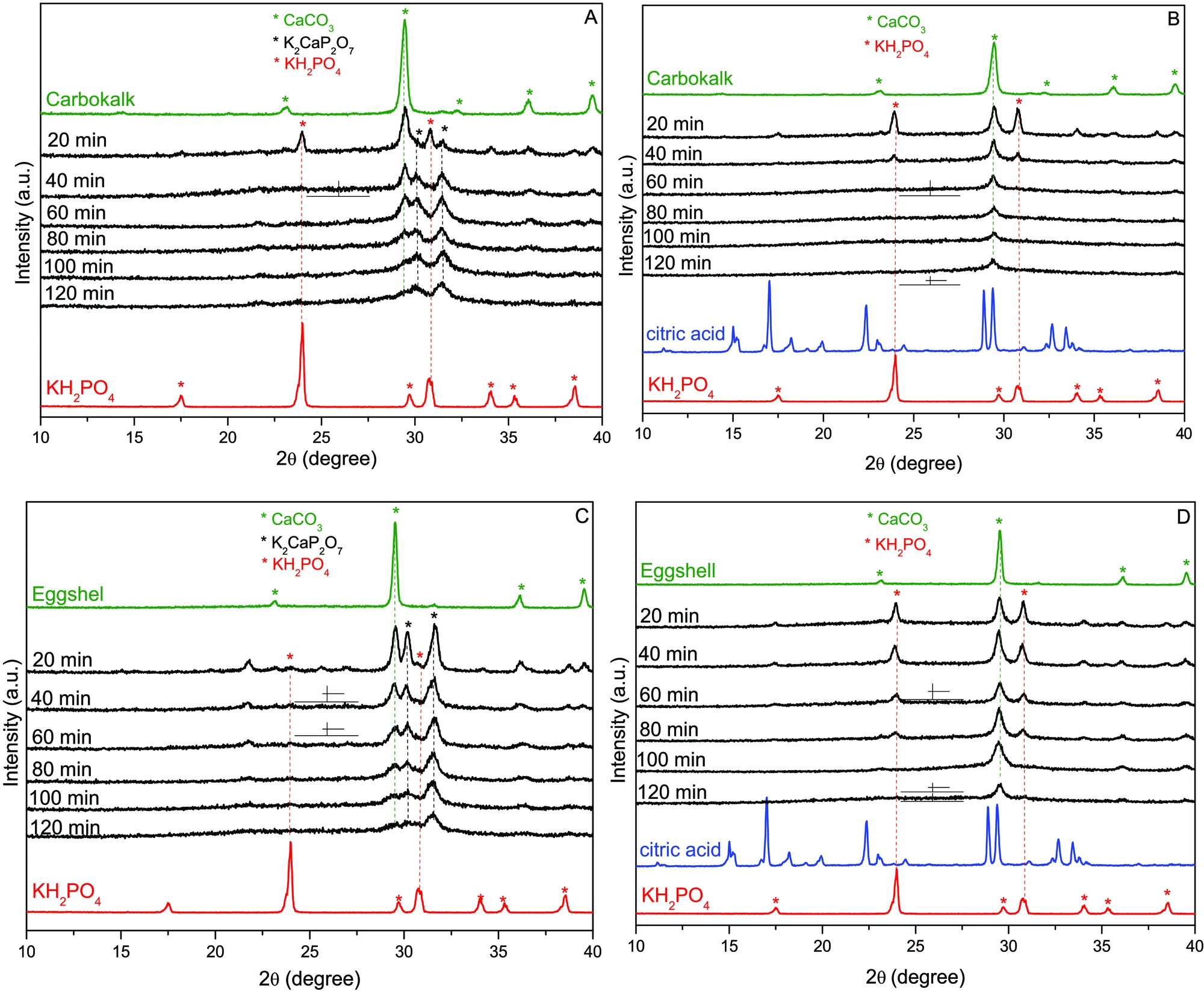
XRD diffractograms of the precursors (eggshell, Carbokalk, KH_2_PO_4_, and citric acid) and milled samples obtained after 20, 40, 60, 80, 100, and 120 min: (A) Carbokalk and KH_2_PO_4_, without citric acid; (B) Carbokalk, KH_2_PO_4_, and citric acid; (C) eggshell and KH_2_PO_4_, without citric acid; and (D) eggshell, KH_2_PO_4_, and citric acid.

On the other hand, with the addition of citric acid in the mixture, no new phase formation was observed (Figure 2B,D). For the Carbokalk CaCO_3_ precursor, the KH_2_PO_4_-related peaks disappeared only after 60 min of milling, whereas for the eggshell precursor, disappearance occurred after 100 min. In both cases, the main peak at 29.5° was not completely consumed, and its enlargement may be attributed to particle size reduction. This behavior suggests that the addition of citric acid generates an alternative and yet unidentified reaction pathway, likely leading to the formation of amorphous phosphates with some residual CaCO_3_. Based on XRD data, 100 min of milling time was selected for further characterization and greenhouse experiments, and the samples have been named according to Table 1. Based on the nominal power of the milling device and processing time (30 Hz, 100 min, 10 g), the estimated specific energy input is 33.4 Wh g^–1^, which is higher than values reported for larger-scale mechanochemical systems. This limitation is therefore acknowledged at laboratory scale. When considered together with the CO_2_ release associated with CaCO_3_ transformation, these results highlight important environmental trade-offs that remain unresolved at this stage. Therefore, a comprehensive life cycle assessment (LCA) will be necessary to properly evaluate the overall sustainability and industrial viability of the proposed process.

**Table 1:** Codes, Physicochemical Characterization of Milled Samples and CaCO_3_-Based Precursors^a^

Sample	Code	phosphorus%	potassium%	calcium%	NP%*	RE**	TNP%***
Carbokalk+KH_2_PO_4_	1	12.71	15.67	18.88	0.55	1	0.55
Carbokalk+KH_2_PO_4_+CA	2	12.40	15.20	15.57	0.30	1	0.30
Eggshell+KH_2_PO_4_	3	12.70	16.10	19.7	5.25	1	5.25
Eggshell+KH_2_PO_4_+CA	4	11.20	14.07	17.73	0.37	1	0.37
Carbokalk precursor	carbokalk	0.905	0.055	31.90	56.44	1	56.44
Eggshell precursor	eggshell	0.079	0.084	33.50	66.67	0	0

a *NP: neutralizing power; **RE: reactivity (1 particles <0.3 mm; 0 particles >2 mm); ***TNP: total neutralizing power (NPxRE).

The nutrient contents presented in Table 1 show that, except for phosphorus differences between the precursors, all milled samples exhibited very similar levels of phosphorus, potassium, and calcium. However, the total neutralization power (TNP) was very different from the neutralization power (NP) and reactivity (RE) of each material. Although eggshell presented the highest NP, the RE was negligible (considering the particle size distribution), resulting in a TNP of 0%. Carbokalk is a powdery feedstock material with 100% of particle size smaller than 0.3 mm and an NP of 56.44, which leads to a TNP of 56.44%. The results indicate that both NPs and TNPs are dependent on the CaCO_3_ content in the materials. The low NP values identified for milled samples suggest that even in low amounts, residual CaCO_3_ is present in all samples. Among the milled samples, sample 3 presents the highest NP and TNP values. Based on this, although the milled samples exhibit a much lower TNP than the CaCO_3_ precursors, the proposed materials are particularly suitable for acidic soils, as they reduce the risk of overliming. Moreover, it is important to highlight that the NPs and TNPs for samples with citric acid may be underestimated due to the influence of this component in determining the acidity correction parameters, according to the methodology used.

FTIR spectra showed that both CaCO_3_-based materials exhibited similar profiles, with characteristic carbonate bands.^27^ Carbokalk also presented some additional bands related to the organic matter (OM) and phosphate groups. This also highlights the beneficial effect of the CaCO_3_-based materials containing residual macronutrients, such as phosphorus, and natural biomass, which is an important factor for soil fertility. Moreover, all milled samples presented the characteristic bands of calcium phosphate, similar to hydroxyapatite groups,^28^ which demonstrate the mechanochemical transformation of the precursors into the desired structures. There was no significant change in the spectral profile when citric acid was added, suggesting that even in amorphous form, K–Ca phosphates are still present in the resulting materials (Figure 3). Moreover, the main carbonate bands are still present, corroborating the presence of residual CaCO_3_-based materials in all final materials, as previously verified by XRD.

**Figure 3:**
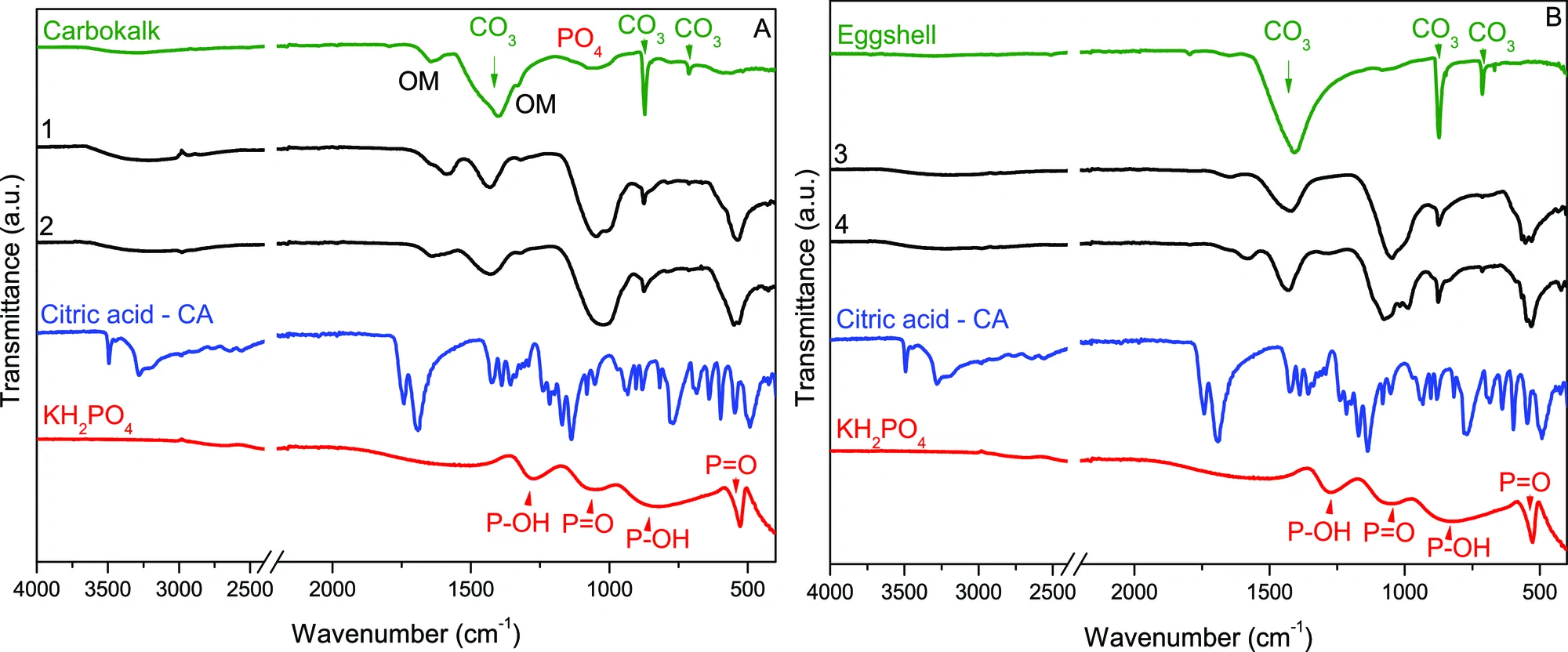
FTIR spectra of the precursors (eggshell, Carbokalk, KH_2_PO_4_, and citric acid) and milled samples: (A) Carbokalk + KH_2_PO_4_ (code 1) and Carbokalk + KH_2_PO_4_ + citric acid (code 2); and (B) eggshell + KH_2_PO_4_ (code 1)­and eggshell + KH_2_PO_4_ + citric acid (code 2). OM = organic matter.

Thermogravimetric analysis revealed three main thermal events (Figure 4A). The first is associated with structural physiosorbed water.^29^ All milled samples and Carbokalk exhibited a water content of approximately 6%, whereas the eggshell precursor contains significantly less, around 1% (Figure 4B). The following thermal event between 200–600 °C is attributed to organic matter decomposition,^30^ which eggshell also shows the lowest organic matter content at 5.8%. The greater mass loss (between 200–600 °C) observed in samples 2 and 4 is attributed to the addition of citric acid prior to milling. The mass loss during the second decomposition step in the CaCO_3_-based material and in milled samples may also be related to structural water release. In the final thermal decomposition step, corresponding to the conversion of CaCO_3_ into CaO and CO_2_, there is a significant reduction in carbonate content.^31^ This is attributed to the mechanochemical reaction with KH_2_PO_4_, as confirmed by XRD analysis and supported by the decrease in Total Neutralization Power. Although the mechanochemical process aims to convert carbonate into less soluble phosphate phases, this reaction pathway intrinsically involves partial CO_2_ release. While this does not affect the nutrient-release behavior, it should be considered in a comprehensive life cycle assessment of the material, as CO_2_ generation represents an environmental trade-off associated with the mechanochemical route. Nevertheless, as a perspective, this drawback can be mitigated by integrating controlled CO_2_ capture during processing, enabling subsequent treatment or conversion pathways aimed at emission reduction. Table S3 shows CaCO_3_ mass loss quantified per kg product.

**Figure 4:**
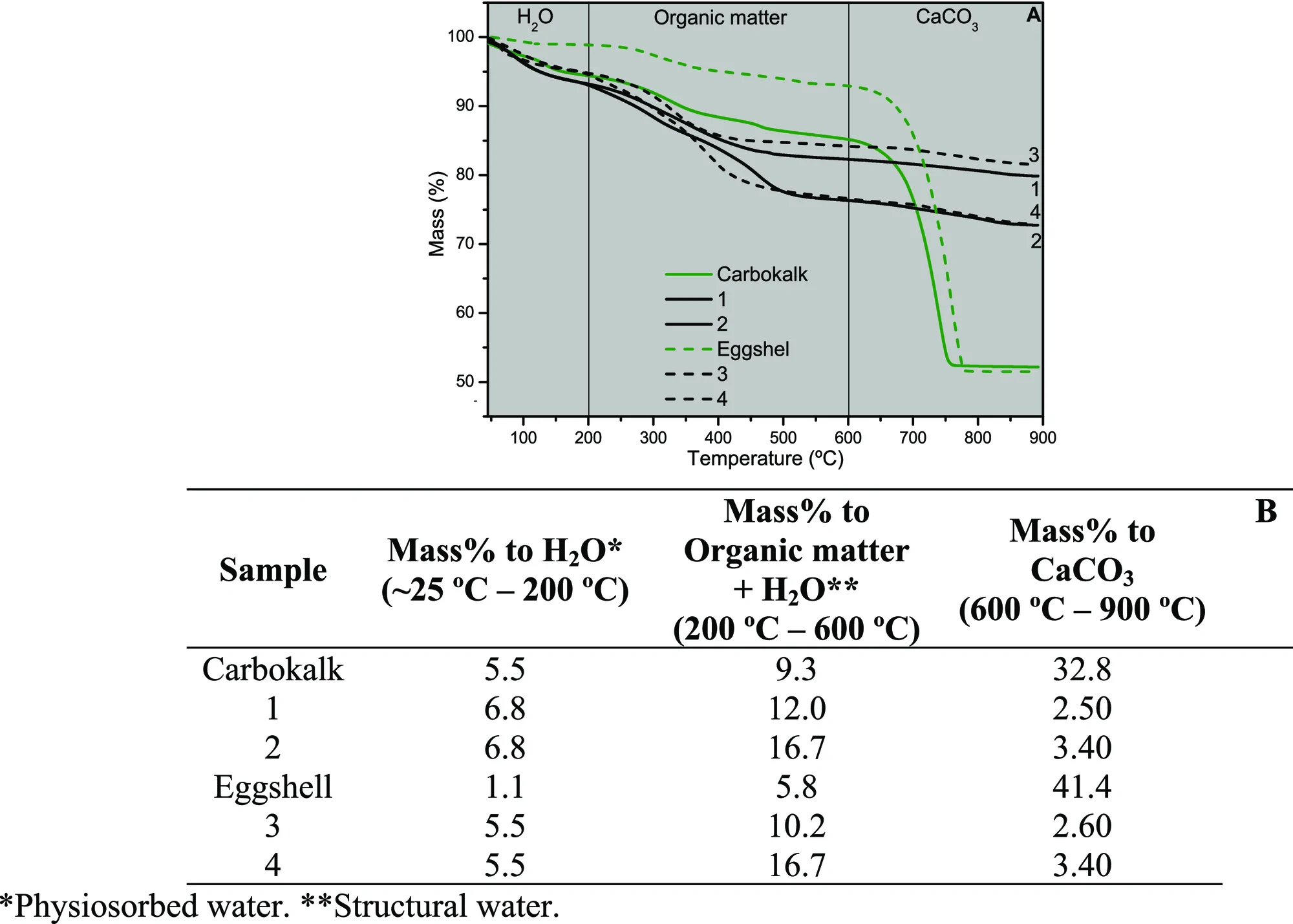
Thermogravimetric analysis of CaCO_3_-based precursors and milled samples (A). Mass loss % composition according to TGA curves (B). Sample codes: 1: Carbokalk+KH_2_PO_4_; 2: Carbokalk+KH_2_PO_4_+CA; 3: Eggshel+KH_2_PO_4_; 4: Eggshel+KH_2_PO_4_+CA. .

The ^13^C NMR spectrum (Figure 5A,B) of Carbokalk shows the presence of inorganic carbonate, in agreement with previous reports.^17^ For sample 1, minor chemical shift variations were observed compared with the CaCO_3_ precursor, Carbokalk, suggesting modifications in the carbon environments after the reaction. In the sample treated with citric acid (sample 2), the characteristic signals of the acid show enhanced intensities, overlapping with the precursor signals. However, the citric acid signals are shifted, which was reported in the literature as potentially related to chelation, generating calcium citrate.^32^ The ^13^C NMR spectrum of eggshell confirms the presence of organic matter, in agreement with the studies developed by Zujovic.^33^ Signals in the 10–70 ppm region can be attributed to residual lipids and proteins. In the carbonyl region (160–180 ppm), resonances can be attributed to the carbonyl groups of the peptide bonds and a lower intensity signal at about 170 ppm to the inorganic carbonate ion (CaCO_3_).^33^ In the spectrum of the sample 3 product, chemical shift changes are observed in comparison with eggshell, indicating modifications in the carbon environments after the reaction. An additional resonance at 169.2 ppm is assigned to inorganic carbonate.^17^ In sample 4, the same behavior as observed for sample 2 was detected, with the citric acid signals prevailing and overlapping those of the original matrix.

**Figure 5:**
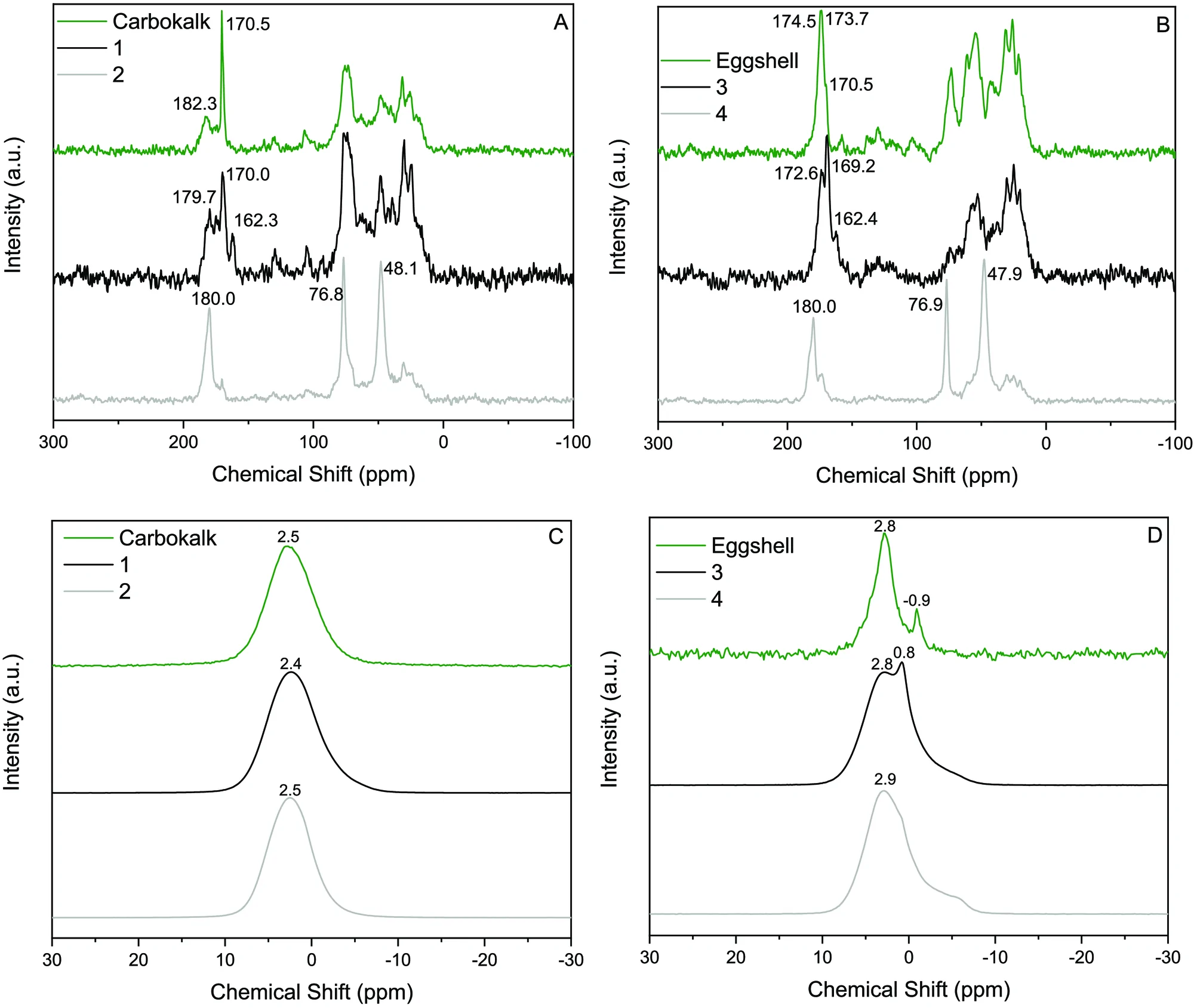
^13^C NMR MAS spectra of calcium carbonate precursors (Carbokalk and Eggshell) and milled samples (A and B, respectively). 31P MAS NMR spectra of calcium carbonate precursors (Carbokalk and Eggshell) and milled samples (C and D, respectively). Sample codes: 1: Carbokalk+KH_2_PO_4_; 2: Carbokalk+KH_2_PO_4_+CA; 3: Eggshel+KH_2_PO_4_; 4: Eggshel+KH_2_PO_4_+CA.

The ^31^P NMR spectrum (Figure 5C,D) of carbokalk, eggshell, and all reaction analogs displays signals in the 2.8–2.9 ppm region, consistent with phosphate environments analogous to hydroxyapatite.^34^ For eggshell, the signals are in agreement with those previously reported in the literature for natural phosphate-based systems, such as chicken eggshell.^33^ After reaction, additional signals around 0.8 ppm suggest the presence of amorphous or secondary phases, which may be associated with secondary phosphate phases (e.g., monetite-type (K_3_CaH­(PO_4_)_2_)), although definitive assignment is not possible.^35^ The peak broadening observed in both sample 1 and sample 3 indicates the coexistence of different phosphorus chemical environments, possibly associated with structural heterogeneity or local disorder within the reaction products. Moreover, such broadening may also be related to the degree of hydration of the samples and the presence of multiple coordination environments.^34^ This behavior suggests that, although the main phase resembles hydroxyapatite, other phosphorus chemical environments cannot be excluded.

### Nutrient Percolation in Sand Soil Substrate and Preliminary Laboratory Simulation Experiments on Nutrient Release

The nutrient percolation behavior in the soil substrate provides proof-of-concept effects regarding the slow-release character of the fertilizer (Figure 6). As expected, KH_2_PO_4_ exhibited a faster percolation of phosphorus and potassium compared to all milled samples, with phosphorus reaching approximately 60% percolation by the second day and approaching 100% after 4 days. Still, when analyzing KH_2_PO_4_, a similar percolation pattern was also observed for potassium.

**Figure 6:**
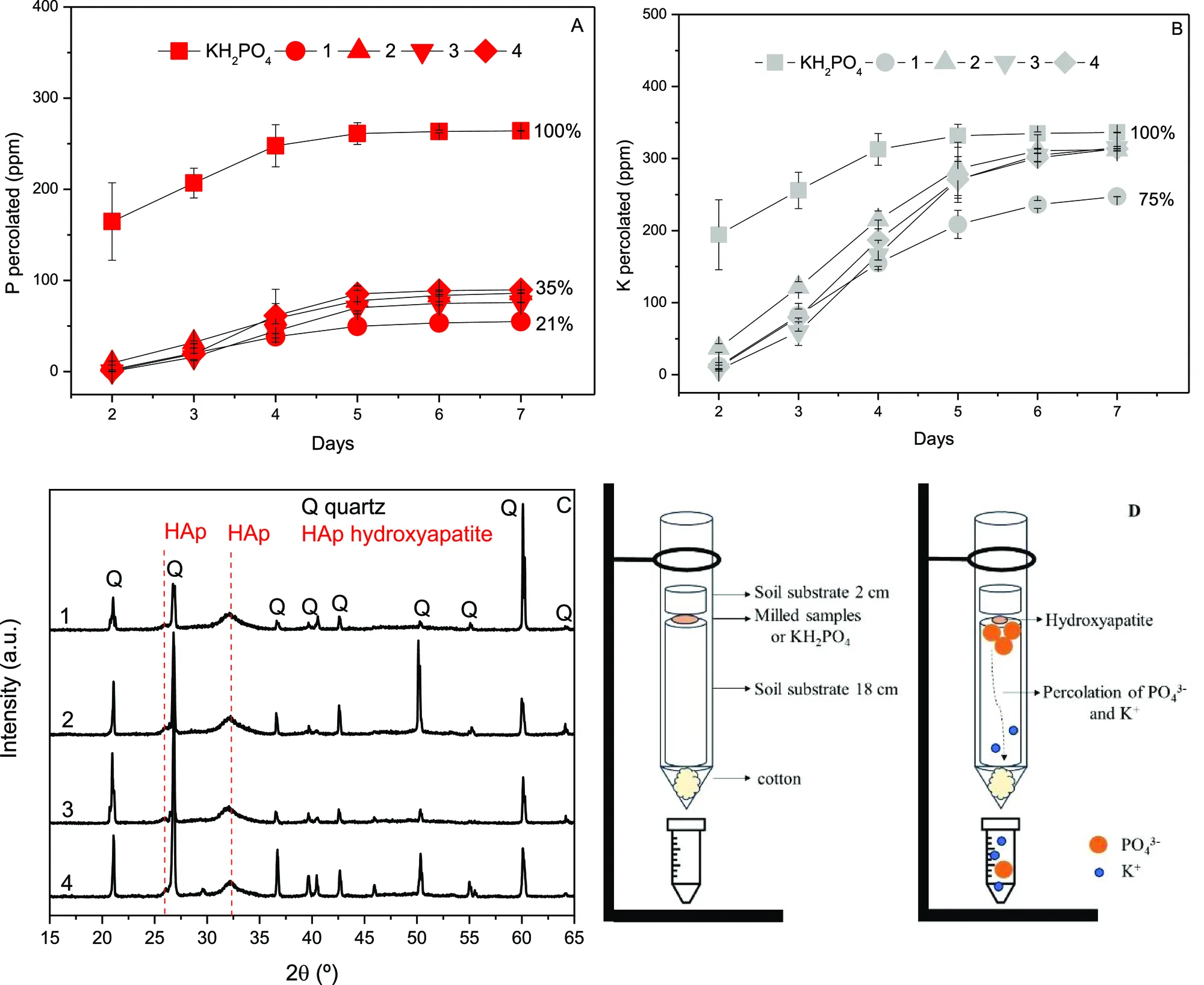
Phosphorus (A) and potassium (B) percolation curves of the precursors KH_2_PO_4_ and milled samples. XRD pattern of the residual samples collected after percolation experiments (C). Sample codes: 1: Carbokalk+KH_2_PO_4_; 2: Carbokalk+KH_2_PO_4_+CA; 3: Eggshel+KH_2_PO_4_; 4: Eggshel+KH_2_PO_4_+CA. Nutrient percolation schematic representation (D).

All milled samples presented similar percolation behavior for both phosphorus and potassium. At the beginning of the experiment (2 days), between 0.2 and 4% of the P content had percolated, increasing to 21-35% by the end (7 days). These results prove that milled samples have higher P retention in the more superficial layers of the soil, ensuring better phosphorus management in the soil. For *K*, the percolation behavior differed from that of phosphorus, with 0.2 to 10% released at the beginning of the experiment, indicating an initial retention of K compared to that of the precursor KH_2_PO_4_. However, with the exception of milled sample 1 that reached only 75% potassium percolation, all other milled samples reached about 100% potassium percolation at the end of the experiment. This is associated with the different interactions between K and Ca compared to P and Ca, in the latter tending to form calcium phosphates of lower solubility.

The results of preliminary laboratory simulation experiments on nutrient release demonstrated a clear reduction in K and P release when compared to the soluble precursor KH_2_PO_4_. While KH_2_PO_4_ reached ∼100% nutrient release within the first 30 min, potassium from the synthesized fertilizers reached complete release only after 24 h. Phosphorus exhibited a markedly slower release, reaching approximately 30% even after 7 days. Samples prepared with the addition of citric acid showed a faster initial release at 30 min compared to those synthesized without citric acid; however, after this initial period, the release profiles tended to stabilize and become similar (Figure S1). Therefore, the influence of citric acid appears to be restricted mainly to the early stages of interaction between the fertilizer and the aqueous or soil solution, without compromising the overall slow-release behavior observed over longer times.

XRD analysis of the residual samples after the percolation experiments corroborates the observed percolation behavior (Figure 6C). Quartz is identified as the predominant crystalline phase in all residual samples, originating from the washed sand used as the substrate.^36^ In addition, hydroxyapatite diffraction patterns are detected, representing a relevant long-term phosphorus source.^37^ These findings support the hypothesis that the main percolation mechanism involves the gradual release of potassium and phosphorus over approximately 5 days. After this period, most of the potassium content is percolated, whereas at least 65% of the phosphorus remains in the upper soil layers, thus remaining accessible to the root system architecture (Figure 6D).

Moreover, these experimental results show that the addition of citric acid to the milled mixture does not significantly affect the overall percolation behavior, despite XRD data indicating the predominance of amorphous phosphate phases and preliminary nutrient-release experiments suggesting differences during the initial release period (30 min). This indicates that structural differences induced by citric acid mainly influence early-stage dissolution kinetics, while the overall nutrient release over longer times is governed by the formation of low-solubility Ca–P phases common to both systems. Thus, it is hypothesized that a cooperative mechanism governs nutrient release, involving the gradual dissolution of amorphous and poorly crystalline Ca–K phosphate phases, followed by the formation of more stable hydroxyapatite-like phases. Given the apparent convergence toward similar Ca–P phases upon interaction with the soil solution, this mechanism appears to be largely independent of the initial degree of crystallinity, which helps explain the comparable percolation behavior observed for samples prepared with and without citric acid.

From the kinetic model fitting of both nutrients, the percolation data for the highly soluble KH_2_PO_4_ precursor exhibited good agreement with both pseudo-first-order and pseudo-second-order models, reflecting its rapid and nonrestrictive dissolution in water. In contrast, the milled samples can be fitted only by the pseudo-first-order model; this behavior is consistent with a more gradual and controlled release profile, although no specific mechanism can be definitively assigned based on these phenomenological models. The distinct kinetic behavior of the mechanochemically treated samples reflects a modulation of nutrient solubility induced by the formation of Ca–P-containing phases and/or amorphous phosphate structures (Figures S2–S4). Moreover, comparison of the pseudo-first-order fits for the precursor and the milled samples (especially sample code 1) shows a decrease in the kinetic constant (*k*_1_), *k*l_1KH2PO4_: 1.58 day^–1^ and *k*_1code1_: 0.93 day^–1^, respectively, consistent with the experimental percolation data (Table S4). The gradual release observed may be associated with the transformation of metastable calcium phosphate phases into more stable forms, such as hydroxyapatite, although this transformation was only qualitatively identified in the present study. Additionally, more complex and/or complementary kinetic mechanisms cannot be ruled out.

### Greenhouse Trials

The plant dry matter production from the greenhouse experiments is shown in Figure 7. In general, compared to milled samples (sample codes 1–4), the positive controls (sample codes 5 and 6, respectively) produced higher average above-ground dry matter in the first and second harvests. In the third harvest, however, the opposite trend was observed, particularly in the treatment with 200 mg of P dm^–3^.

**Figure 7:**
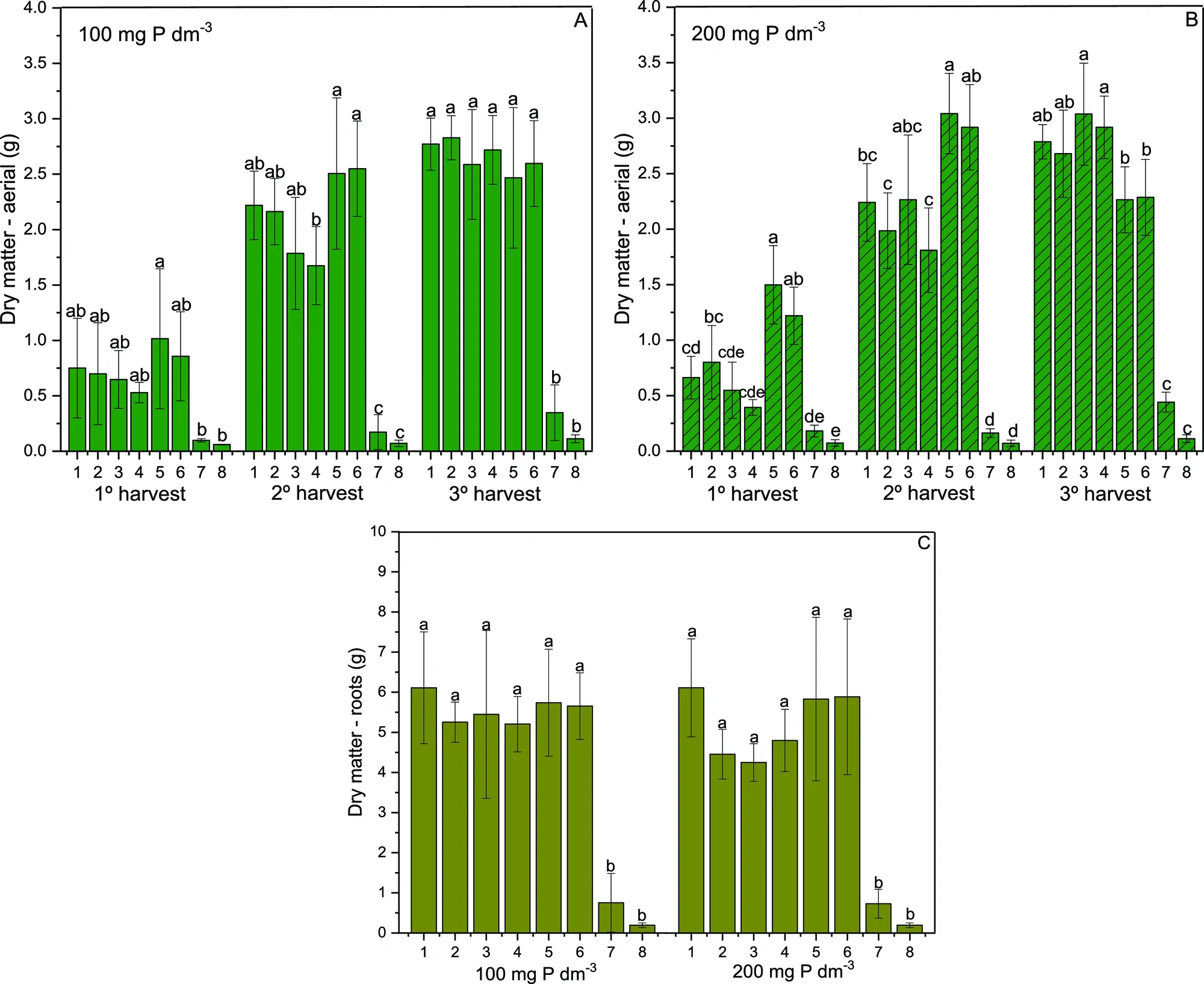
Dry matter production of above-ground biomass considering 100 mg P dm^–3^ (A) and 200 mg P dm^–3^ (B) during three harvests. Dry matter production of roots considering 100 mg P dm^–3^ and 200 mg P dm^–3^ at the third/final harvest (C). Samples code: 1: Carbokalk+KH_2_PO_4_; 2: Carbokalk+KH_2_PO_4_+CA; 3: Eggshel+KH_2_PO_4_; 4: Eggshel+KH_2_PO_4_+CA; 5: KH_2_PO_4_; 6: TSP; 7: Bayovar rock; 8: unfertilized control. Bar chartssharing the same letter are not statistically different between treatments within each harvest and dose level (ANOVA and Tukey’s test, *p* < 0.05).

The temporal trend may reflect the onset of mineral depletion in the soil substrate, as exemplified by the results of the third harvest in the positive controls. However, the average values for the milled samples indicate a slight increase in the above-ground dry matter production from the second to the third harvest, which may be associated with improved nutrient management in the substrate and, consequently, greater nutrient uptake by the plants. Moreover, the comparison between milled samples and the negative control (sample code 7) shows that natural rock phosphates usually present lower nutrient release behavior, which compromises dry matter production when nutrient delivery and uptake processes are not adequate. The low efficiency of the negative control can also be verified from the root dry matter production (Figure 7C). Milled samples and positive controls present similar root dry matter production; however, the negative control results were more than five times lower, corroborating the above-ground dry matter production. These behaviors may be related to the low-nutrient plant bioavailability of natural rock phosphates, which, although an important source of phosphorus fertilization, are more suitable for long-term crop fertilization or even perennial crops.

The additional controls consisting of a simple physical mixture of CaCO_3_-based material and KH_2_PO_4_ (without milling—sample codes 9 and 10) show that the performance, regarding above-ground dry matter production, of this physical blend closely matches that of the KH_2_PO_4_ control (sample code 5), indicating that mere mixing of the precursors does not reproduce the behavior obtained after mechanochemical processing (Figure S5).

The analysis of the P, K, and Ca contents in the produced dry matter (Figure 8) confirmed the previously proposed behavior in dry matter production. The application of 100 mg P dm^–3^ of milled samples showed an almost linear P uptake behavior. In comparison, in the application of 200 mg P dm^–3^, there was a slight increase in uptake from the first to the final harvest. In contrast, the positive controls exhibited a completely different behavior, with maximum uptake occurring at the second harvest, followed by a significant decrease at the final harvest. This suggests a more efficient P management using the proposed milled samples. Regarding K, the milled samples showed an increased content in dry matter at the second harvest, followed by a slight decrease at the final harvest. Although similar to the milled samples, the positive controls present lower values at the final harvest. This is an important experimental observation, since K is usually highly mobile in soil, indicating that the calcium–potassium phosphate materials produced could also be a viable alternative for improving sustainable K fertilization.

**Figure 8:**
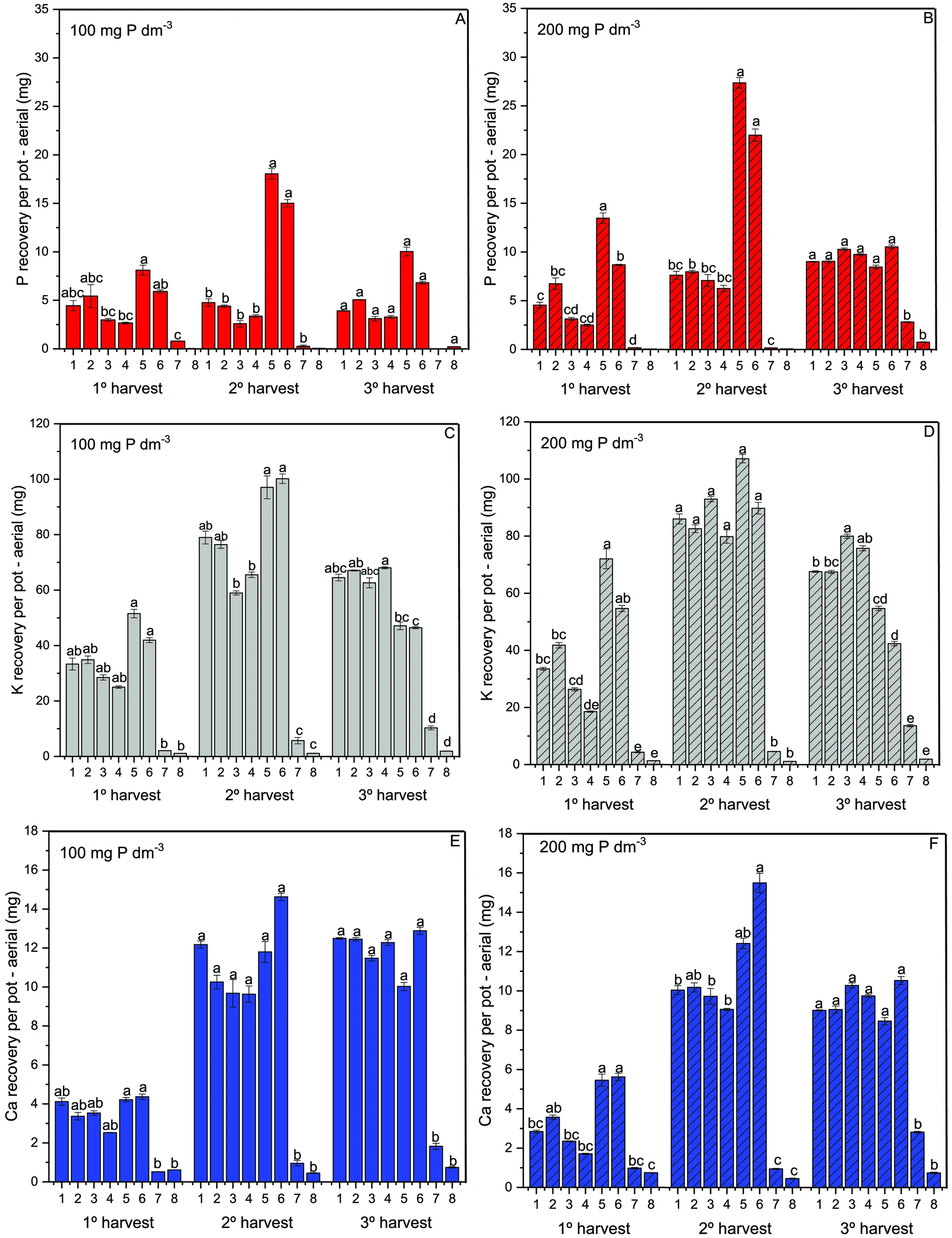
Nutrient uptake considering 100 mg P dm^–3^ and 200 mg P dm^–3^ during three harvests. Phosphorus uptake (A, B); potassium uptake (C, D); and calcium uptake (E, F). Sample codes: 1: Carbokalk+KH_2_PO_4_; 2: Carbokalk+KH_2_PO_4_+CA; 3: Eggshel+KH_2_PO_4_; 4: Eggshel+KH_2_PO_4_+CA; 5: KH_2_PO_4_; 6: TSP; 7: Bayovar rock; 8: unfertilized control. Bar charts sharing the same letter are not statistically different between treatments, within each harvest and dose level (ANOVA and Tukey’s test, *p* < 0.05).

Finally, the Ca analysis generally showed that the milled samples increased the uptake at the second harvest and maintained an almost linear trend at the final harvest. The positive controls exhibited a similar but less pronounced behavior, resembling that observed for the P uptake. Thus, it is evident that the Ca behavior follows P uptake, indicating that its availability is directly dependent on fertilizer dissolution as proposed.

Analogous to the observation for above-ground dry matter production, the additional controls consisting of a simple physical mixture of the CaCO_3_-based material and KH_2_PO_4_ (without milling—sample codes 9 and 10) also revealed that the plant uptake of P, K, and Ca was very similar to that observed for the KH_2_PO_4_ control (sample code 5) (Figure S6). In other words, the physical mixture shows a marked increase in nutrient uptake in the second harvest followed by a decrease in the third harvest, which differs from the behavior observed for the milled samples and demonstrates that mere physical mixing of the precursors is not sufficient to ensure proper nutrient management.

The greenhouse trials conducted in this research already demonstrate the promising potential of the milled materials for improved nutrient management, particularly with regard to the delivery of P and K under controlled conditions. These results provide solid proof of concept and justify the continued development of these materials. As a future perspective, multiseason field trials will be important to validate their performance under real soil conditions, considering natural variability in climate, soil chemistry, microbial, and crop dynamics. Such studies will allow a more comprehensive assessment of agronomic efficiency but lie beyond the scope of this work. Additionally, a comprehensive life cycle assessment, including energy demand, greenhouse gas emissions, and downstream environmental impacts, was beyond the scope of this work and should also be addressed in future studies.

## Conclusion

Efficient management of macronutrients such as potassium (K) and phosphorus (P) is essential to sustain agricultural productivity while addressing environmental concerns related to nutrient losses. In this study, we demonstrated that KH_2_PO_4_ can react with CaCO_3_ under mechanochemical activation, using different sources, more specifically: spent lime (Carbokalk) from the sugar beet industry and eggshells. The resulting materials are predominantly characterized by calcium–potassium phosphate phases. However, when citric acid is incorporated into the milling mixture, no crystalline phosphate phases are detected, which may be associated with disrupted structural organization and/or partial Ca complexation, resulting in predominantly amorphous phosphate products along with residual CaCO_3_.

All milled materials exhibited similar percolation behavior and reduced nutrient release compared to KH_2_PO_4_ under the experimental conditions applied. This reduced solubility suggests that the milled samples may favor retention of K and P closer to the application zone, potentially improving nutrient availability and management in agricultural systems. The release behavior can be interpreted as arising from the interaction of metastable Ca–K phosphate phases and/or amorphous phosphates with water, potentially evolving toward more stable Ca–P phases such as hydroxyapatite, which functions as a long-term nutrient source.

Greenhouse experiments with *Brachiaria brizantha* across three successive harvests supported these observations. The soluble positive controls (KH_2_PO_4_ and TSP) showed a peak in biomass production and nutrient uptake in the second harvest, followed by a marked decline in the third. In contrast, plants receiving the milled samples exhibited linear or gradually increasing responses throughout the experiment. This pattern is consistent with a more progressive nutrient release, which may help reduce nutrient losses and improve fertilizer use efficiency. These results demonstrate the potential of mechanochemically produced Ca–K phosphate materials as a proof-of-concept toward more efficient fertilization strategies.

## Supplementary Material



## Notes

Macronutrient and micronutrient contents, CaCO_3_ mass loss, kinetic parameters, analyses of variance, and trace metal concentrations; preliminary nutrient-release experiments; kinetic analyses of phosphorus and potassium release; dry matter production; and nutrient uptake data from plant experiments (Tables S1–S15) (Figures S1–S6) (PDF)

## References

[1] Scholz R. W., Ulrich A. E., Eilittä M., Roy A. (2013). Sustainable use of
phosphorus: A finite resource. Sci. Total Environ..

[2] Vejan P., Khadiran T., Abdullah R., Ahmad N. (2021). Controlled release
fertilizer: A review on developments, applications and potential in
agriculture. J. Controlled Release.

[3] YAHAYA S. M., MAHMUD A. A., ABDULLAHI M., HARUNA A. (2023). Recent advances in
the chemistry of nitrogen, phosphorus and potassium as fertilizers
in soil: A review. Pedosphere.

[4] Hart M. R., Quin B. F., Nguyen M. L. (2004). Phosphorus
Runoff from Agricultural
Land and Direct Fertilizer Effects: A Review. J. Environ. Qual..

[5] Chien S. H., Prochnow L. I., Tu S., Snyder C. S. (2011). Agronomic and environmental
aspects of phosphate fertilizers varying in source and solubility:
an update review. Nutr. Cycl. Agroecosyst..

[6] Machado R. C., Valle S. F., Chao I. R. S., Ribeiro C. (2024). Valorization of Alum
Sludge Waste through Zeolite Synthesis for Sustainable Fertilizer
Production. Mater. Res..

[7] Timilsena Y. P., Adhikari R., Casey P., Muster T., Gill H., Adhikari B. (2015). Enhanced efficiency
fertilisers: a review of formulation
and nutrient release patterns. J. Sci. Food
Agric..

[8] Wang H., DeLisio J. B., Wu T., Wang X., Zachariah M. R. (2018). One-step
solvent-free mechanochemical synthesis of metal iodate fine powders. Powder Technol..

[9] Borges R., Wypych F. (2019). Potential Slow Release Fertilizers
and Acid Soil Conditioners
Obtained by One-Pot Mechanochemical Activation of Chrysotile:Cement
Roofing Sheets with K2HPO4. J. Braz. Chem. Soc..

[10] Borges R., Baika L. M., Grassi M. T., Wypych F. (2018). Mechanochemical conversion
of chrysotile/K2HPO4 mixtures into potential sustainable and environmentally
friendly slow-release fertilizers. J. Environ.
Manage..

[11] Borges R., Soares Giroto A., Klaic R., Wypych F., Ribeiro C. (2021). Mechanochemical
synthesis of eco-friendly fertilizer from eggshell (calcite) and KH2PO4. Adv. Powder Technol..

[12] Borges R., Brondi M., Elias A. M., Farinas C. S., Ribeiro C. (2024). Mechanochemical
recycling of cellulose multilayer carton packages to produce micro
and nanocellulose from the perspective of techno-economic and environmental
analysis. J. Environ. Manage..

[13] Borges R., Giroto A. S., Guimarães G. G.
F., Reis H. P. G., Farinas C. S., Ribeiro C. (2022). Asbestos cement waste treatment through
mechanochemical process with KH2PO4 for its utilization in soil pH
correction and nutrient delivery. Environ. Sci.
Pollut. Res..

[14] Chen X., Yang S., Wen X. (2026). Simulation of sugar
beet growth in
saline-alkali land: Controlling the synergistic effect of aluminum
sulfate irrigation on photosynthesis. Eur. J.
Agron..

[15] Gupta S., Kant K., Kaur N., Jindal P., Ali A., Naeem M. (2025). Nano-calcium applications in modern agriculture: A review. Plant Nano Biol..

[16] Sahadat
Hossain M., Shaikh M. A. A., Ahmed S. (2023). Synthesis of gypsum
fertilizer from waste eggshells for a sustainable environment. Mater. Adv..

[17] Borges R., Soares M. B., Pollo M. P., Alleoni L. R. F., Giroto A. S., Gonçalves M., Müller M., Jablonowski N. D., Ribeiro C. (2025). Zinc and Cadmium Remediation in Contaminated Soil by
CaCO3-Biochar Material from Sugar Beet Industry. Water, Air, Soil Pollut..

[18] Merlin A., Rosolem C. A., He Z. (2016). Non-labile
phosphorus acquisition
by Brachiaria. J. Plant Nutr..

[19] Borges R., Sotiles A. R., Giroto A., Guimarães G., Wypych F., Jablonowski N., Ribeiro C. (2022). Mechanochemical Activation
of Elemental Sulfur Increases Its Bioavailability in the Forage Species *Brachiaria* Production. ACS Agricult.
Sci. Technol..

[20] Marín-Benito J., Brown C. D., Herrero-Hernández E., Arienzo M., Sánchez-Martín M. J., Rodríguez-Cruz M. S. (2013). Use of
raw or incubated organic wastes as amendments in reducing pesticide
leaching through soil columns. Sci. Total Environ..

[21] Owens D. S., Johnson G. V. (1996). Fertilizer nutrient leaching and nutrient mobility:
A simple laboratory exercise. J. Nat. Resour.
Life Sci. Educ..

[22] Martins L. E. C., Monteiro F. A., Pedreira B. C. (2015). Photosynthesis and
Leaf Area of *Brachiaria brizantha* in Response to
Phosphorus and Zinc
Nutrition. J. Plant Nutr..

[23] Almeida E. M. d., Difante G. d. S., Montagner D. B., Araújo A. R. d., Macedo M. C. M., Gurgel A. L. C., Monteiro G. O. d. A., Santana J. C. S. (2024). The supply of phosphate fertilizers
affects the uptake of macronutrients and the productivity of *Brachiaria* cultivars during the establishment period. J. Plant Nutr..

[24] Bosi C., Sentelhas P. C., Huth N. I., Pezzopane J. R. M., Andreucci M. P., Santos P. M. (2020). APSIM-Tropical Pasture: A model for
simulating perennial tropical grass growth and its parameterisation
for palisade grass (Brachiaria brizantha). Agricult.
Syst..

[25] Han K., Seo S., Lee S., Kim M. J. (2022). A Process for removing toxic heavy
metals to produce the high purity NH4H2PO4 and KH2PO4 from a crude
phosphoric acid. J. Hazard. Mater. Adv..

[26] Al-Jaroudi S. S., Ul-Hamid A., Mohammed A.-R. I., Saner S. (2007). Use of X-ray powder
diffraction for quantitative analysis of carbonate rock reservoir
samples. Powder Technol..

[27] Mehta N., Gaëtan J., Giura P., Azaïs T., Benzerara K. (2022). Detection
of biogenic amorphous calcium carbonate (ACC)
formed by bacteria using FTIR spectroscopy. Spectrochim. Acta, Part A.

[28] Prekajski M., Mirković M., Todorović B., Matković A., Marinović-Cincović M., Luković J., Matović B. (2016). Ouzo effect—New simple nanoemulsion
method for
synthesis of strontium hydroxyapatite nanospheres. J. Eur. Ceram. Soc..

[29] Mohamed E. A., Ahmed H. M., Altalhi A. A., Al-Shamiri H. A. S., Negm N. A. (2025). Highly efficient and rapid removal
of Congo red dye
from textile wastewater using facile synthesized Mg/Ni/Al layered
double hydroxide. Sci. Rep..

[30] Jalu R. G., Chamada T. A., Kasirajan Dr. R. (2021). Calcium
oxide nanoparticles synthesis
from hen eggshells for removal of lead (Pb­(II)) from aqueous solution. Environ. Challenges.

[31] Bahrom H., Goncharenko A. A., Fatkhutdinova L. I., Peltek O. O., Muslimov A. R., Koval O. Yu., Eliseev I. E., Manchev A., Gorin D., Shishkin I. I., Noskov R. E., Timin A. S., Ginzburg P., Zyuzin M. V. (2019). Controllable Synthesis
of Calcium Carbonate with Different
Geometry: Comprehensive Analysis of Particle Formation, Cellular Uptake,
and Biocompatibility. ACS Sustainable Chem.
Eng..

[32] Borges R., do Amaral L. F. M., Ribeiro V. P., de Oliveira-
Paiva C. A., Wypych F., Ribeiro C. (2023). A Mechanochemical Route
to Magnesium/Calcium
Citrate Formation for Chrysotile/Asbestos Cement Correct Disposal
as Agricultural Input. Waste Biomass Valorization.

[33] Zujovic Z., Bowmaker G. A. (2024). On the Structure and Role of Avian Eggshells: A ^31^P, ^1^H, and ^13^C Solid-State NMR Study. J. Agric. Food Chem..

[34] Yu Y., Guo H., Pujari-Palmer M., Stevensson B., Grins J., Engqvist H., Edén M. (2019). Advanced solid-state
1H/31P NMR characterization of pyrophosphate-doped calcium phosphate
cements for biomedical applications: The structural role of pyrophosphate. Ceram. Int..

[35] Yu Y., Stevensson B., Pujari-Palmer M., Guo H., Engqvist H., Edén M. (2019). The Monetite
Structure Probed by Advanced Solid-State
NMR Experimentation at Fast Magic-Angle Spinning. Int. J. Mol. Sci..

[36] Tavares L. R. C., Junior J. F. T., Costa L. M., da Silva Bezerra A. C., Cetlin P. R., Aguilar M. T. P. (2020). Influence of quartz powder and silica
fume on the performance of Portland cement. Sci. Rep..

[37] Hashemi N., Vaezi Z., Masoumi S., Mohammadi S. S., Mahdavian R., Sedghi M., Redl H., Banerjee A., Weidinger A., Naderi-Manesh H. (2022). Temperature dependent physicochemical
characteristics, antibacterial and cytotoxic potential of iron quantum
cluster templated hydroxyapatites. Ceram. Int..

[38] Godfray H. C. J., Beddington J. R., Crute I. R., Haddad L., Lawrence D., Muir J. F., Pretty J., Robinson S., Thomas S. M., Toulmin C. (2010). Food security: The challenge of feeding
9 billion people. Science.

[39] FAO - Food and Agriculture Organization of the United Nations . The future of food and agriculture - Trends and challenges. FAO, Rome, Italy, 2017 https://openknowledge.fao.org/server/api/core/bitstreams/2e90c833-8e84-46f2-a675-ea2d7afa4e24/content (accessed 2026–01–20).

[40] Brekalo I., Martinez V., Karadeniz B., Orešković P., Drapanauskaite D., Vriesema H., Stenekes R., Etter M., Dejanović I., Baltrusaitis J., Užarević K. (2022). Scale-up of
agrochemical urea–gypsum cocrystal synthesis using thermally
controlled mechanochemistry. ACS Sustainable
Chem. Eng..

[41] DeSutter T. M., Godsey C. B. (2010). Sugar-beet-processing lime as an amendment for low
pH soils. Commun. Soil Sci. Plant Anal..

[42] Borges R., Giroto A. S., Ohrem B., Beckmann S., Ademi A., Boeckem V., Bochmann H., Müller-Linow M., Lenz H., Ribeiro C., Wojciechowski T., Jablonowski N. D., Postma J. A. (2025). Optimizing cassava growth with localized
struvite application: Root proliferation and fertilization efficiency. Agronomy.

[43] Majaron V. F., Vellani C. L., Martins Junior A. A.
F., Soares S. M., Putti F. F., Innocentini M. D. M., Bernardi A. C. C., Polito W. L., Ribeiro C., Bortoletto-Santos R. (2025). Role of nanocomposite structure in
polyurethane coatings for slow-release fertilizers: A case study with
Brachiaria brizantha. ACS Agricult. Sci. Technol..

[44] Yi H., Gao B., Zhang X., Liang Y., Zhang J., Su J. (2024). Application
of waste eggshells elevates phytoremediation efficiency of Pb–Zn
mine-contaminated farmland and mitigates soil greenhouse gas emissions:
A field study. J. Environ. Manage..

