# Supplementary material for: Evaluating the Slow-Release Fertilizer Effects of Calcium Potassium Phosphates from Mechanochemically Treated Calcium Carbonate Materials with KH2PO4
Source: ACS Omega. 2026 Oct 17;11(43):66077–90. doi: 10.1021/acsomega.5c12414 (PMC13737994; doi:10.1021/acsomega.5c12414)
Supplement: Supplementary file 1 [file ao5c12414_si_001.pdf]

Support Information

Evaluating the slow-release fertilizer effects of calcium potassium phosphates from mechanochemically treated calcium-carbonate materials with KH_2PO_4

Roger Borges[1*]; Amanda S. Giroto[2,3]; Stella F. Valle[4,5], Benedict Ohrem[3]; Tainára Orlando[1]; Paloma Neves de Oliveira[1], Nicolai D. Jablonowski[3]; Caue Ribeiro[5]*.

[1] Federal Technological University of Paraná, Medianeira, PR, 85884-000, Brazil.

[2] Federal University of São Paulo, Science and Technology Institute, 12231-280 São José dos Campos, SP, Brazil.

[3] Institute of Bio- and Geosciences, IBG-2: Plant Sciences, Forschungszentrum Jülich GmbH, 52425 Jülich, Germany.

[4] Lorena School of Engineering (EEL), University of São Paulo (USP), 12602-810, Lorena, SP, Brazil.

[5] Embrapa Instrumentation, XV de Novembro Street, 1452, 13560-970, São Carlos, SP, Brazil.

Table S1. Macronutrient content.

Nutrient %	Milled Samples				Controls		
	CKP	CKPAC	EKP	EKPAC	KH_2PO_4	TSP	Bayovar rock
**N**	0.28	0.24	0.63	0.47	0	0	0
**K**	15.67	15.2	16.1	14.07	27.1	0	0.202
**Ca**	18.88	15.57	19.7	17.73	0	10	30.6
**Mg**	0.505	0.415	0.176	0.164	0	0	0.328
**S**	0.474	0.39	0.416	0.376	0	0	1.96
**C**	5.17	7.31	4.4	7.12	0	0	0
**P**	12.71	12.4	12.7	11.2	21.4	17.5	12.6

Table S2. Micronutrient fertilization.

Micronutrient	Dose (mg Kg^{-1} of soil)	Source
B	1.4	H_3BO_3
Cu	3.5	$CuSO_4$
Mn	4.5	$MnCl_2.4H_2O$
Zn	0.12	$ZnSO_4.7H_2O$
Mo	0.7	$(NH_4)_6Mo_7O_{24}.4H_2O$

Table S3 - $CaCO_3$ mass loss quantified per kg product.

Sample	Mass (CO_2 per kg of product) to $CaCO_3$ (600 ºC – 900 ºC)
Carbokalk	328
1	25.0
2	34.0
Eggshell	414
3	26.0
4	34.0

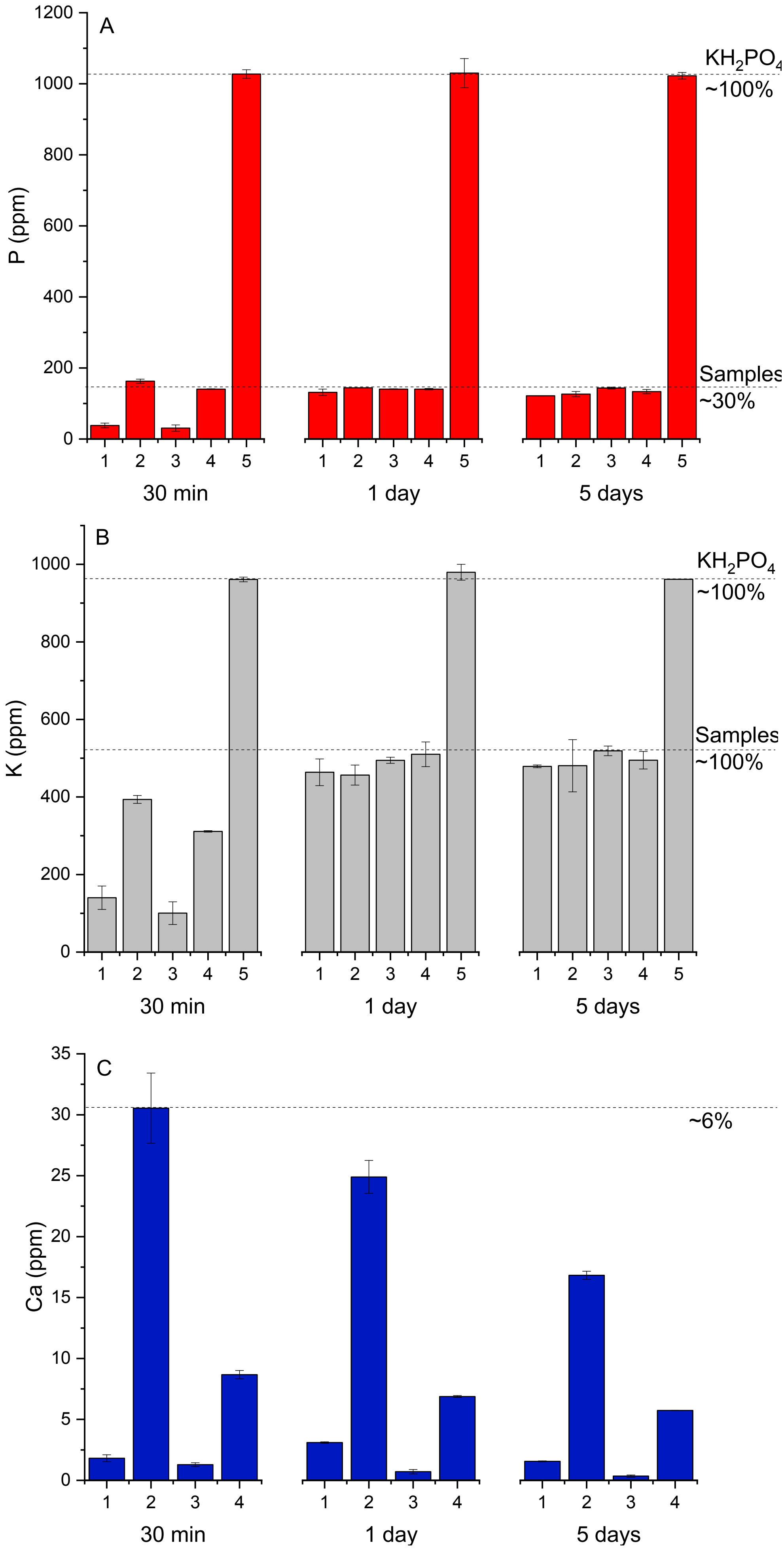

Fig. S1. Preliminary simulation experiments on nutrient release: (A) phosphorus, (B) potassium, and (C) calcium.

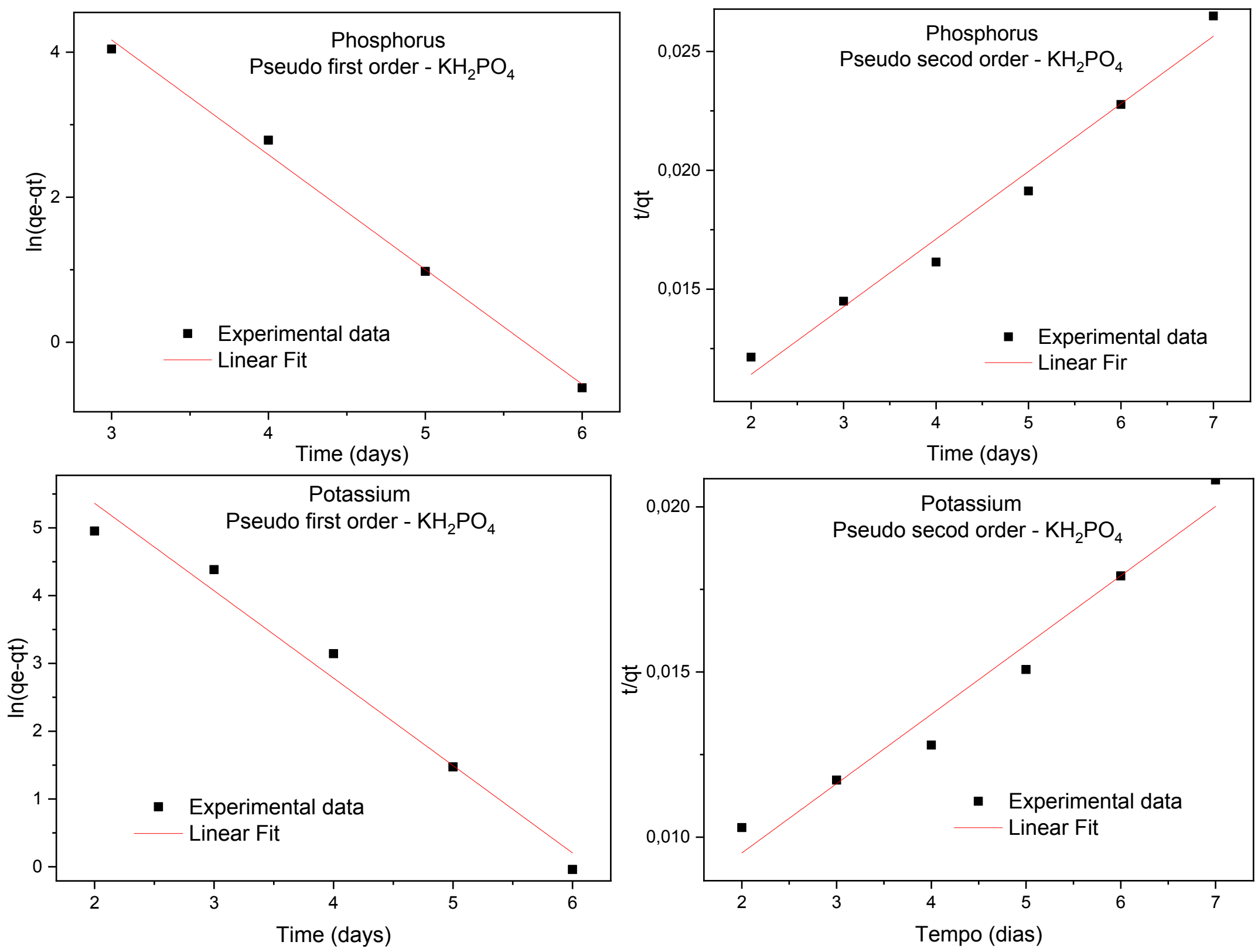

Fig. S2. Linear fit and experimental data of pseudo-first-order and pseudo-second-order of phosphorus and potassium percolation experiments of KH_2PO_4.

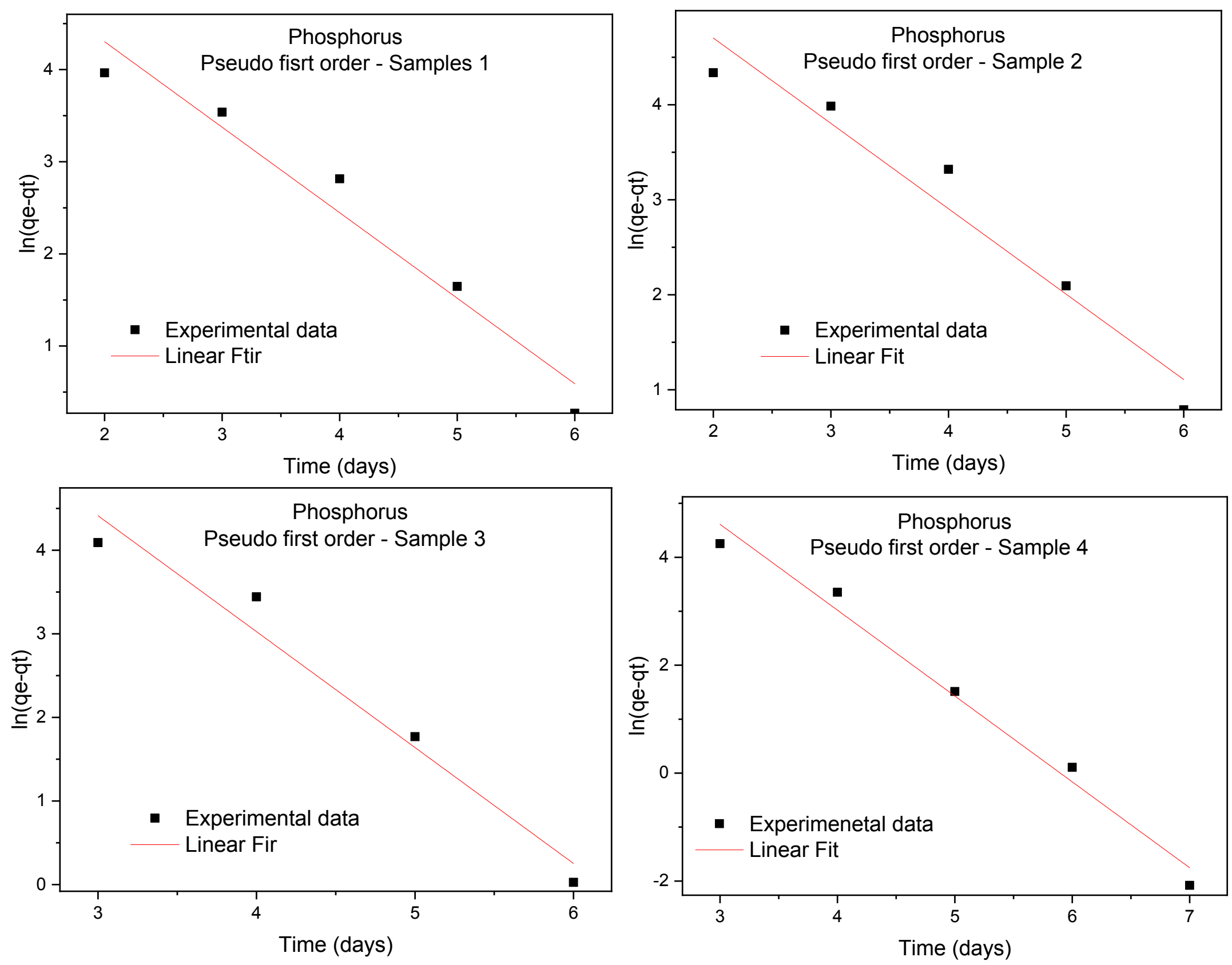

Fig. S3. Linear fit and experimental data of pseudo-first-order of phosphorus percolation experiments of milled samples. Samples code: 1: Carbokalk+KH_2PO_4; 2: Carbokalk+KH_2PO_4+CA; 3: Eggshel+KH_2PO_4; 4: Eggshel+KH_2PO_4+CA.

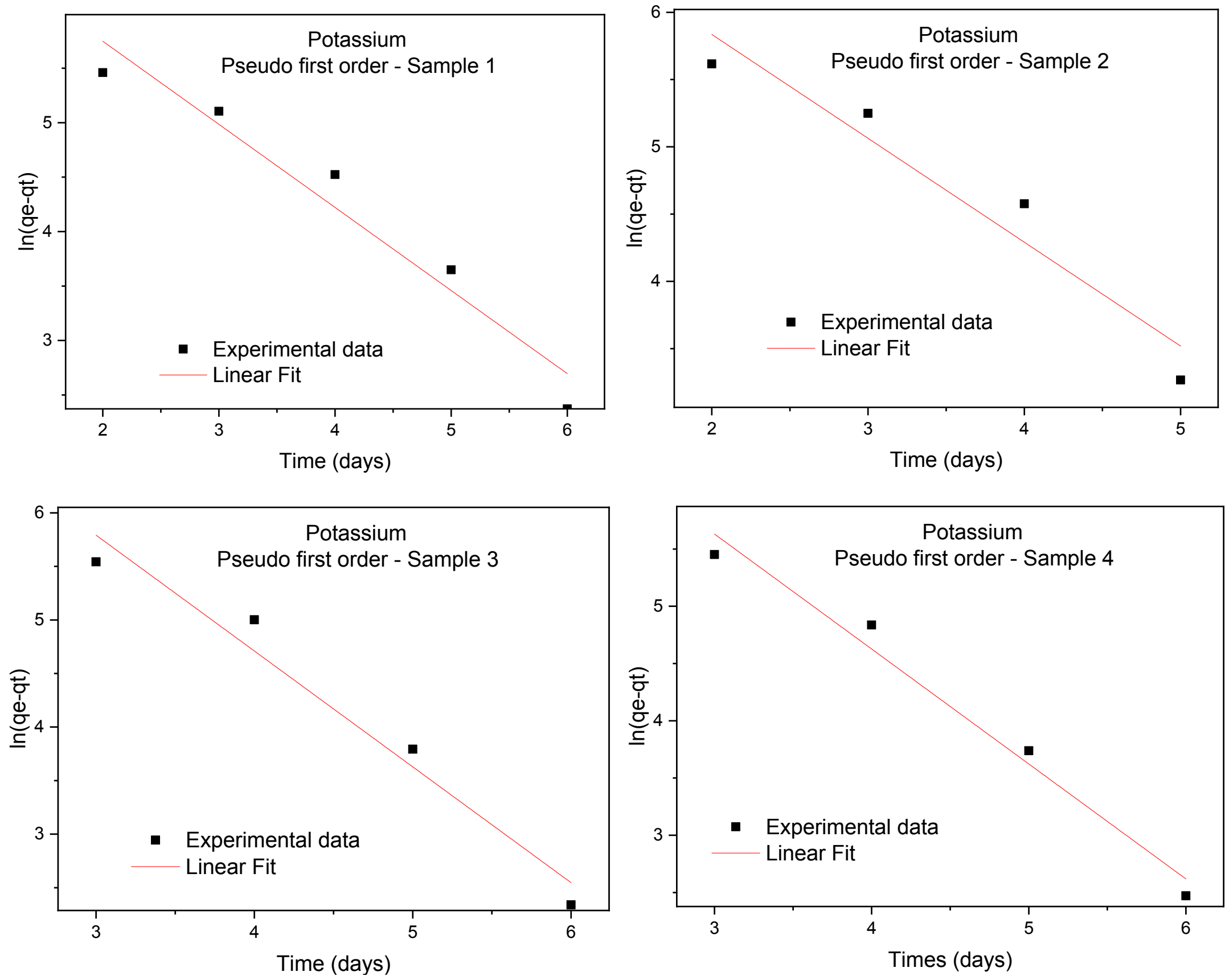

Fig. S4. Linear fit and experimental data of pseudo-first-order of potassium percolation experiments of milled samples. Samples code: 1: Carbokalk+KH_2PO_4; 2: Carbokalk+KH_2PO_4+CA; 3: Eggshel+KH_2PO_4; 4: Eggshel+KH_2PO_4+CA.

Table S4. Kinetic parameters of phosphorus and potassium release fitted to pseudo-first-order and/or pseudo-second-order models for the KH_2PO_4 precursor and milled samples.

	Phosphorus				Potassium			
	Pseudo-first-order		Pseudo-second-order		Pseudo-first-order		Pseudo-second-order	
Sample	k_1 (day^{-1})	R^2	k_2 (L mg^{-1} day^{-1})	R^2	k_1 (day^{-1})	R^2	k_2 (L mg^{-1} day^{-1})	R^2
KH_2PO_4	1.58	0.99	$1.42.10^{-2}$	0.98	1.29	0.97	$9.49.10^{-3}$	0.96
1	0.93	0.94			0.76	0.93		
2	1.08	0.97			0.77	0.89		
3	1.39	0.95			1.08	0.95		
4	1.42	0.97			1.00	0.97		

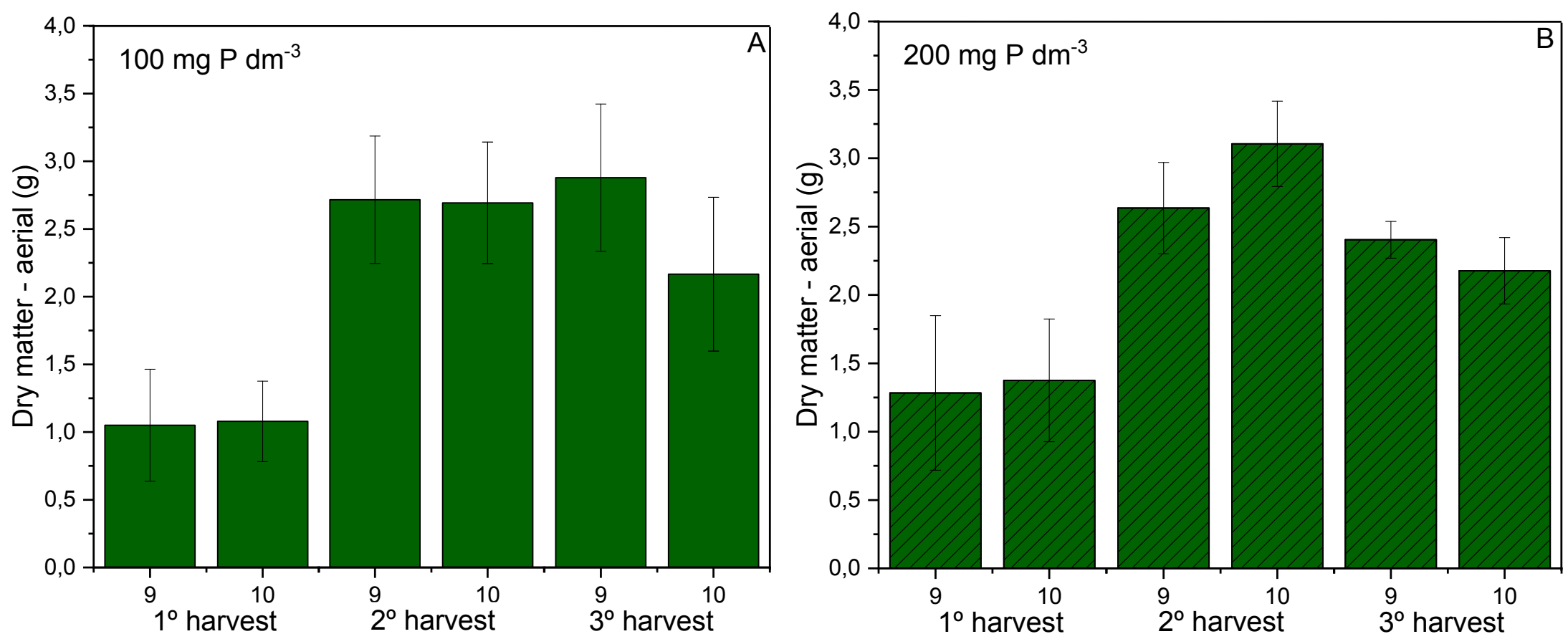

Fig. S5. Dry matter production of above ground biomass considering 100 mg P dm^{-3} (A) and 200 mg P dm^{-3} (B) during three harvests. Samples code: 9: unmilled Carbokalk+KH_2PO_4; 10: unmilled Eggshel+KH_2PO_4.

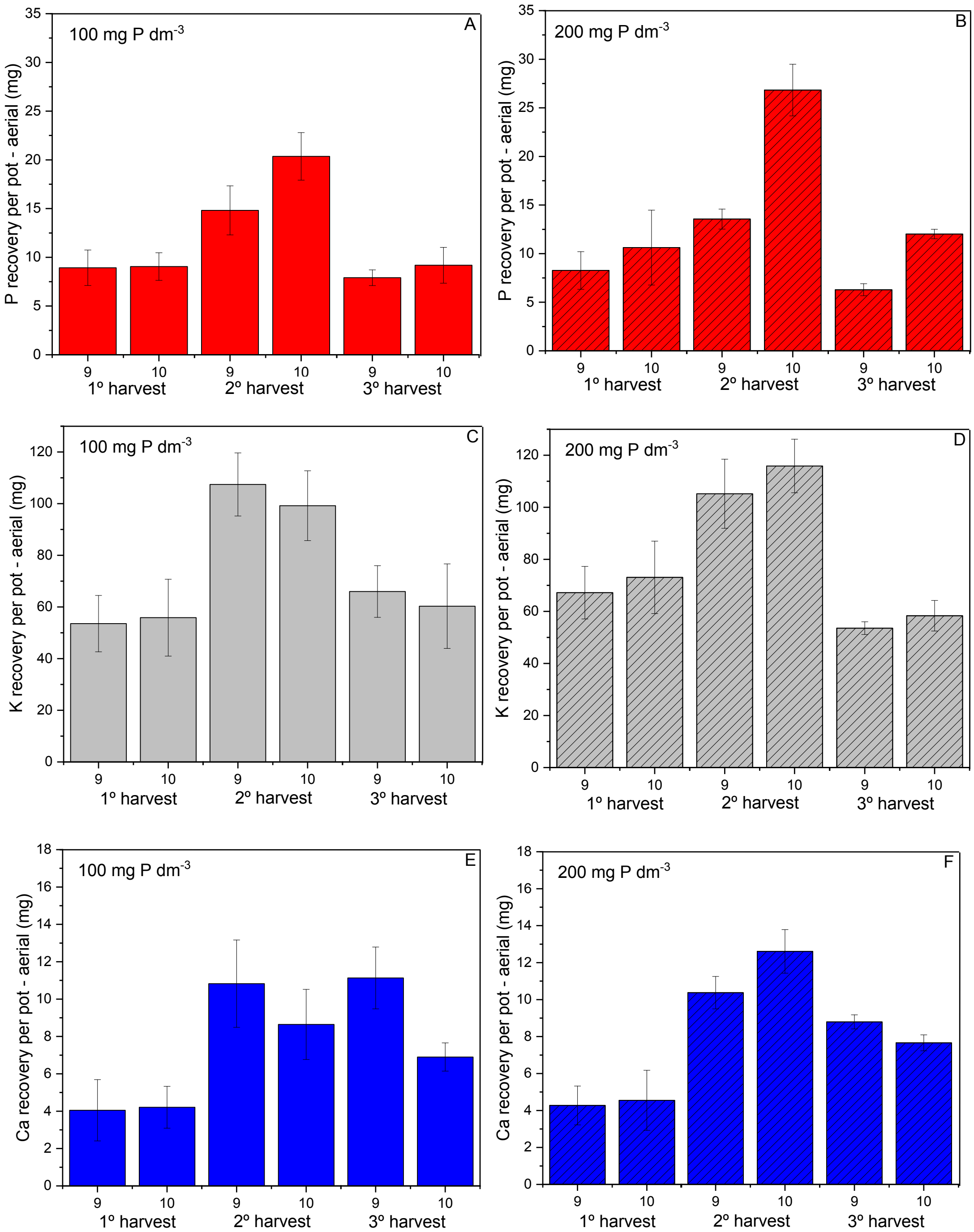

Figure S6 - Nutrient uptake considering 100 mg P dm^{-3} and 200 mg P dm^{-3} during three harvests. Phosphorus uptake (A) and (B); Potassium uptake (C) and (D); and Calcium uptake (E) and (F). Samples code: 9: unmilled Carbokalk+KH_2PO_4; 10: unmilled Eggshel+KH_2PO_4.

Table S5 - Analysis of variance for dry matter production under 100 mg P dm^{-3}

**Source of Variation**	**Sum of Squares**	**df**	**F-value (F)**	**p-value (PR(>F)*)**
C(Treatment)	63,73597925	7	64,59410353	1,50x10^{-31}
C(Cut)	40,89026715	2	145,0428099	2,79x10^{-28}
C(Treatment):C(Cut)	13,69055803	14	6,937442032	2,71x10^{-09}
Residual	12,12250017	86		

*Probability associated with the ANOVA F-test for each source of variation; values below 0.05 indicate rejection of the null hypothesis and confirm significant differences among at least one group mean relative to the others.

Table S6 – Analysis of of variance for dry matter production under 200 mg P dm^{-3}

Source of Variation	Sum of Squares	df	F-value (F)	p-value (PR(>F))
C(Treatment)	37,61892	2	223,3522	$8,8x10^{-35}$
C(Cut)	73,63759	7	124,9153	$2,78x10^{-42}$
C(Treatment):C(Cut)	20,6507	14	17,51543	$1,59x10^{-19}$
Residual	7,242433	86		

Table S7 - Analysis of variance for root dry matter production under 100 mg P dm^{-3}

Source of Variation	Sum of Squares	df	F-value (F)	p-value (PR(>F))
C(Treatment)	194,0645	7	22,42312	$6,71x10^{-10}$
Residual	34,61865	28		

Table S8 - Analysis of variance for root dry matter production under 200 mg P dm^{-3}

Source of Variation	Sum of Squares	df	F-value (F)	p-value (PR(>F))
C(Treatment)	171,5055	7	23,31518	$4,27\ x10^{-10}$
Residual	29,42383	28		

Table S9 – Analysis of variance for nutrient uptake under 100 mg P dm^{-3}

Source of Variation	Sum of Squares	df	F-value (F)	p-value (PR(>F))
C(Treatment)	94124,49	2	1,585163	0,210342
C(Cut)	290434,9	7	1,397501	0,215554
C(Treatment):C(Cut)	979882,6	14	2,357476	0,007575
Residual	2790786	94		

Table S10 - Analysis of variance for nutrient uptake under 200 mg P dm^{-3}

Source of Variation	Sum of Squares	df	F-value (F)	p-value (PR(>F))
C(Treatment)	486,7048	2	36,95633	$1,25x10^{-12}$
C(Cut)	3306,397	7	71,73154	$2,5x10^{-35}$
C(Treatment):C(Cut)	1402,994	14	15,21882	$1,24x10^{-18}$
Residual	632,147	96		

Table S11 - Analysis of variance for nutrient uptake under 100 mg K dm^{-3}

Source of Variation	Sum of Squares	df	F-value (F)	p-value (PR(>F))
C(Treatment)	22094,96	2	56,20537	$6,93x10^{-17}$
C(Cut)	68576,88	7	49,84187	$3,13x10^{-29}$
C(Treatment):C(Cut)	14993,2	14	5,448548	$1,48x10^{-07}$
Residual	18869,34	96		

Table S12 - Analysis of variance for nutrient uptake under 200 mg K dm^{-3}

Source of Variation	Sum of Squares	df	F-value (F)	p-value (PR(>F))
C(Treatment)	26210,44	2	109,4241	$1,74x10^{-25}$
C(Cut)	85830,96	7	102,3799	$1,15x10^{-41}$
C(Treatment):C(Cut)	22406,64	14	13,36342	$6,57x10^{-17}$
Residual	11497,47	96		

Table S13 - Analysis of variance for nutrient uptake under 100 mg Ca dm^{-3}

Source of Variation	Sum of Squares	df	F-value (F)	p-value (PR(>F))
C(Treatment)	999,7331827	2	123,39	$2,94x10^{-27}$
C(Cut)	1546,076491	7	54,52039	$1,08x10^{-30}$
C(Treatment):C(Cut)	353,419071	14	6,231433	$1,16x10^{-08}$
Residual	388,9065187	96		

Table S14 - Analysis of variance for nutrient under 200 mg Ca dm^{-3}

Source of Variation	Sum of Squares	df	F-value (F)	p-value (PR(>F))
C(Treatment)	721,7944	2	103,0728	$1,25x10^{-24}$
C(Cut)	1254,238	7	51,1732	$1,17x10^{-29}$
C(Treatment):C(Cut)	329,0148	14	6,711939	$2,55x10^{-09}$
Residual	336,1326	96		

Table S15 - Concentrations of trace metals in slow-release fertilizer extracts (mean ± standard deviation, ppm).

Sample	Cu (ppm)		Fe (ppm)		Mn (ppm)		Mo (ppm)	
	Mean	± SD	Mean	± SD	Mean	± SD	Mean	± SD
CKP	0,00635	0,00516	0,0223	0,0123	0,003	0,00156	0,00285	0,00205
CKP-AC	0,0268	0,01259	0,02725	0,00304	0,0015	0,00198	0,00295	0,00262
EKP	0,0134	0,01414	0,01015	0,00799	0,0012	$1,41421E^{-4}$	0,00215	$7,77817E^{-4}$
EKP-AC	0,0228	0,00396	0,00315	0,00332	8,5E-4	0,00106	0,0037	0,00156

SD = Standard deviation.

Note: No detectable levels of Cd, Co, Cr, Hg, Ni, Zn, or Pb were found.
